# Mitochondrial and Sarcoplasmic Reticulum Interconnection in Cardiac Arrhythmia

**DOI:** 10.3389/fcell.2020.623381

**Published:** 2021-01-28

**Authors:** Felipe Salazar-Ramírez, Roberto Ramos-Mondragón, Gerardo García-Rivas

**Affiliations:** ^1^Tecnologico de Monterrey, Escuela de Medicina y Ciencias de la Salud, Cátedra de Cardiología y Medicina Cardiovascular, Monterrey, Mexico; ^2^Department of Pharmacology, University of Michigan Medical School, Ann Arbor, MI, United States; ^3^Department of Internal Medicine, Division of Cardiovascular Medicine, University of Michigan Medical School, Ann Arbor, MI, United States; ^4^TecSalud, Centro de Investigación Biomédica, Hospital Zambrano-Hellion, San Pedro Garza García, Mexico; ^5^TecSalud, Centro de Medicina Funcional, Hospital Zambrano-Hellion, San Pedro Garza García, Mexico

**Keywords:** mitochondria, sarcoplasmic reticulum, arrhythmia, calcium, heart failure, ischemia/reperfusion injury

## Abstract

Ca^2+^ plays a pivotal role in mitochondrial energy production, contraction, and apoptosis. Mitochondrial Ca^2+^-targeted fluorescent probes have demonstrated that mitochondria Ca^2+^ transients are synchronized with Ca^2+^ fluxes occurring in the sarcoplasmic reticulum (SR). The presence of specialized proteins tethering SR to mitochondria ensures the local Ca^2+^ flux between these organelles. Furthermore, communication between SR and mitochondria impacts their functionality in a bidirectional manner. Mitochondrial Ca^2+^ uptake through the mitochondrial Ca^2+^ uniplex is essential for ATP production and controlled reactive oxygen species levels for proper cellular signaling. Conversely, mitochondrial ATP ensures the proper functioning of SR Ca^2+^-handling proteins, which ensures that mitochondria receive an adequate supply of Ca^2+^. Recent evidence suggests that altered SR Ca^2+^ proteins, such as ryanodine receptors and the sarco/endoplasmic reticulum Ca^2+^ ATPase pump, play an important role in maintaining proper cardiac membrane excitability, which may be initiated and potentiated when mitochondria are dysfunctional. This recognized mitochondrial role offers the opportunity to develop new therapeutic approaches aimed at preventing cardiac arrhythmias in cardiac disease.

## Introduction

Arrhythmias can be defined as any disturbance in the normal electrical sequence of the heart. These disturbances may cause the electrical impulse to travel slowly, rapidly, or in an erratic manner. However, few studies have calculated the overall burden of arrhythmias. Incidence has been reported to be about 2.35% in the United Kingdom’s general population (Khurshid et al., 2018). In Mexico, only data for atrial fibrillation (AF) exist, and it is estimated to affect 2% of the general population (Lara-Vaca et al., 2014). These data are consistent with the estimated global rates of approximately 1–4% (Zulkifly et al., 2018). Although it may seem like a low percentage, electrical abnormalities appear in up to 39% of patients with cardiopathies (Vazquez Ruiz de Castroviejo et al., 2005). Within this population, sudden cardiac death constitutes a significant cause of mortality. Sudden cardiac death is defined as when the death of a patient occurs, most commonly by a fatal ventricular arrhythmia, within 1 h of the onset of symptoms when there is a witness or within 24 h of last being seen alive when no witness is available (Adabag et al., 2010). Arrhythmia susceptibility is especially concerning in high-risk populations, such as heart failure (HF) patients, in which the incidence of sudden cardiac death reaches approximately 15% per year; this population continues to grow, as it is the outcome of almost all cardiovascular pathologies (Lee et al., 2011; Srinivasan and Schilling, 2018). Sudden cardiac death has been documented to be responsible for 20–30% of all cardiac deaths worldwide and about 7–18% in the United States (Rodríguez-Reyes et al., 2015). Analyzing the process of arrhythmogenesis at the cellular level is, therefore, of vital importance to better understand the underlying mechanisms that lead to its development and elucidate new potential therapeutic targets to prevent it.

Focal activity and re-entry are proposed as the main mechanisms of cardiac arrhythmia. Re-entry is associated with conduction abnormalities and occurs when a propagating impulse fails to extinguish after normal activation of the heart tissue and re-excites the heart after completion of the refractory period. A reduction in the cardiac impulse’s wavelength, which is a product of the conduction time and refractory period (Smeets et al., 1986; Wijffels et al., 1995), is a determinant of re-entry. Changes in the expression and function of membrane ion channels (termed electrical remodeling) affect the cardiac conduction properties and refractory period, facilitating functional re-entry (Papadatos et al., 2002). The alteration of gap junction-specialized structures that couple myocytes (Kirchhoff et al., 1998; Jansen et al., 2010), the deposition of extracellular matrix components (Verheule et al., 2004; Krul et al., 2015), and cardiac enlargement (Eijsbouts et al., 2003; Vranka et al., 2007) are considered part of the structural remodeling that increases the chances of arrhythmia via anatomical re-entry mechanisms. Triggered activity refers to the impulse initiation resulting from depolarization of the membrane potential after the upstroke phase of the action potential (AP). Afterdepolarizations that occur during the repolarization phase of the AP are called early-afterdepolarizations (EADs), whereas those occurring after completion of the membrane repolarization are known as delayed-afterdepolarizations (DADs). The modulation of membrane ion channels that prolong the AP facilitates the appearance of EADs, whereas the appearance of DADs is commonly associated with altered intracellular Ca^2+^ homeostasis. Both increased Ca^2+^ overload conditions and Ryanodine receptor (RyR2) dysfunction (leakiness) promote spontaneous Ca^2+^ releases from the sarcoplasmic reticulum (SR) and activate the Na^+^/Ca^2+^ exchanger (NCX) in its forward mode. In this operating mode, a net inward Na^+^ current (I_ti_) is present, which depolarizes the membrane and causes DADs. If these DADs are large enough, they can reach the threshold activation of the Na^+^ current and generate a full arrhythmogenic AP. Both EADs and DADs are known as trigged activity and play an important role in initiating cardiac arrhythmias. There is compelling evidence that mitochondria play an important role in the generation of Ca^2+^-triggered arrhythmia, a process that requires an understanding of cellular Ca^2+^ fluxes.

The SR is the organelle in charge of storing and releasing Ca^2+^ into the cytosol. RyR2 opening occurs after a small initial amount of Ca^2+^ passes through L-type Ca^2+^ channels (LTCCs) in the sarcolemma in response to membrane depolarization during the AP (Bers, 2002). The sudden increase in cytosolic Ca^2+^ levels activates the myofibrils in sarcomeres, and contraction occurs. During the diastolic phase, Ca^2+^ is removed from the cytosol, and around 70% of total cytosolic Ca^2+^ is pumped back into the SR (Bassani et al., 1994) by the sarco/endoplasmic reticulum Ca^2+^ ATPase (SERCA). Ca^2+^ is extruded into the extracellular space by NCX activation, which counterbalances the entry of Ca^2+^ through LTCCs. Only a discreet quantity of cytosolic Ca^2+^ is removed by mitochondria (Bers, 2002), which occurs through a Ca^2+^ channel in the inner mitochondrial membrane (IMM) known as mitochondrial Ca^2+^ uniplex (mCU) (previously called the mitochondrial Ca^2+^ uniporter). Mitochondrial Ca^2+^ extrusion is carried out by the mitochondrial NCX, which possesses slow kinetics compared to the mCU, allowing the accumulation of this ion in the mitochondria. The local SR-Mitochondria Ca^2+^ flux is facilitated by anchoring proteins that anchor both organelles (Kohlhaas and Maack, 2013). The dependence on Ca^2+^ import in mitochondrial reactive oxygen species (ROS) production and energetics highlights the importance of coordinated regulation between SR Ca^2+^ fluxes and mitochondrial homeostasis. In this review, we focus on describing the cross signals that occur between these two organelles and how dysregulation of this intertwined signaling may be involved in altered cardiac rhythms.

## The Mitochondria-SR Interconnection: Proximity Enables Crosstalk

**FIGURE 1:**
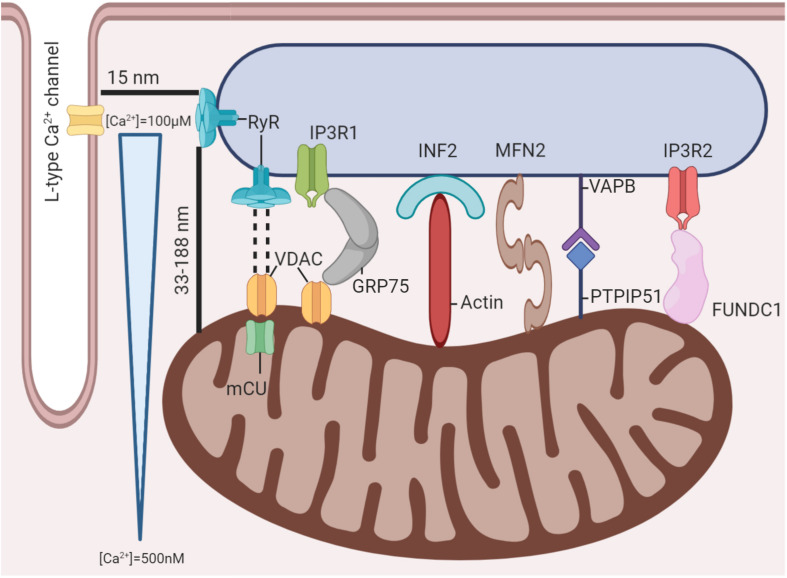
Mitochondria-SR-T tubule microdomain. Several protein structures have been demonstrated to exist between mitochondria and the SR. These structures maintain proximity between the organelles and enable mitochondria to experience significant Ca^2+^ fluctuations with each AP despite the global cardiomyocyte’s Ca^2+^ concentration remaining relatively unchanged.

The structure of the cardiomyocyte has been extensively reviewed (Eisner et al., 2017), and emphasis has been placed on the proximity of T-tubules, where LTCCs reside, with the terminal cisternae of the SR, where RyR2s are more concentrated. The approximate distance between both structures has been calculated to be only about 15 nm (Scriven et al., 2013). This distance is what enables the two structures to react to each other’s activation. This unit, comprised of a T-tubule with its corresponding terminal cisternae of the SR, is called a dyad; it is considered the functional unit of the heart and is in charge of excitation-contraction-coupling (ECC). Nonetheless, because of its proximity, mitochondria could also be regarded as part of this functional unit, as they also play a role in responding to stimulation and producing a proper contractile response. Fluorescence (Friedman et al., 2011) and electron microscopy (Csordás et al., 2006) techniques have been used to reveal physical interactions between these organelles, with protein-like structures linking both membranes. Protein structures between the mitochondria and the SR have been described elsewhere (Lopez-Crisosto et al., 2017; Martinvalet, 2018; Giorgi et al., 2018). Several structures have been found to create bridges between the organelles, securing proximity (Figure 1). The complexity of SR-mitochondria bridging proteins is high, so the removal of individual structures could be compensated for by other components. Describing the interactions of each element found to link both organelles in detail is out of the scope of this review, but a brief description will be provided. The first of such structures is the Ca^2+^ channel inositol-3-phosphate receptor (IP3R), joined to the mitochondrial voltage-dependent anion channel (VDAC) through protein GRP75 (Szabadkai et al., 2006). This communication enables the rapid movement of Ca^2+^ ions from the SR into the mitochondrial intermembrane space when IP3 is released through the protein kinase C (PKC) pathway. Similarly, IP3R2 has been described as binding with the FUN14 domain containing 1 (FUNDC1) to modulate SR Ca^2+^ release (Wu et al., 2017). The VDAC has also been described as having physical interactions with the RyR2, which, coupled with mCU co-localization with the RyR2, helps explain how mitochondrial Ca^2+^ transport is possible (Kohlhaas and Maack, 2013). Other structures involved in maintaining a connection are the SR vesicle-associated membrane protein-associated protein B/C (VAPB), whose function is not fully understood, although it has been shown to regulate Ca^2+^ transport between both organelles (De Vos et al., 2012). The mitochondrial protein tyrosine phosphatase-interacting protein 51 (PTPIP51) seems to have more structural functions (Stoica et al., 2014). The ER-mitochondria encounter structure (ERMES) is a protein complex characterized in yeast that bridges both organelles and has diverse biological functions. Although no homolog structure has been described in mammals, an ortholog of one of its components, PDZ domain-containing protein 8 (PDZD8), was described recently (Giorgi et al., 2018). Other structures, such as Mitofusin2, a protein involved in mitochondrial dynamics, have also been described as being able to form dimers that bridge both organelles (de Brito and Scorrano, 2008), presumably to organize mitochondrial dynamics. Similarly, SR protein inverted formin 2 (INF2) serves as an anchor for actin filaments to reach the mitochondria, thereby providing scaffolding for mitochondrial constriction in mitochondrial dynamics (Korobova et al., 2013; Manor et al., 2015). These connections help maintain the close gap between mitochondria and the SR. Intermyofibrillar mitochondria have been measured as close as 33 nm to the RyR2 in the SR and as far away as 188 nm (Ramesh et al., 1998). This proximity enables the existence of a microdomain where secure communication can take place. For instance, cellular Ca^2+^ concentrations vary from about 100–500 nM globally between resting and peak concentrations in the AP (Bers, 2002). Furthermore, within the dyadic cleft, Ca^2+^ levels can get as high as 100 μM at the periphery when the RyR2 releases Ca^2+^ from the SR, stimulated by LTCCs (Langer and Peskoff, 1996). These high levels of Ca^2+^ are maintained for about 10 ms, affecting a region of about 2 μm, although not in the same concentrations, before descending as Ca^2+^ diffuses to other cellular regions (Cheng and Lederer, 2008). This localization places the mitochondria well within reach of accessing high local Ca^2+^ concentrations. Although 10 ms may not seem very long, this is enough time for Ca^2+^ to be transported through the IMM by the mCU into the mitochondrial matrix. Two models of the mitochondrial response to changes in cytosolic Ca^2+^ levels have been described (O’Rourke and Blatter, 2009). The first one, originally proposed by Crompton (Crompton, 1990), indicates that the mitochondrial Ca^2+^ concentration increases slowly and gradually with a faster AP firing rate until influx and efflux are balanced entirely, and a new steady state is achieved. However, slow changes in the mitochondrial Ca^2+^ concentration may not be able to stimulate ATP production fast enough to meet immediate metabolic needs. Subsequently, beat-to-beat changes in mitochondrial Ca^2+^ are slight, and the energetic requirements of integrated mitochondrial Ca^2+^ transport are marginal. The other model describes mitochondria as having the ability to comprehensively sense rapid cytosolic Ca^2+^ changes, presenting with oscillations on a beat-to-beat basis (O’Rourke and Blatter, 2009). This process would imply that mitochondria can not only rapidly internalize but also extrude Ca^2+^ ions. In consolidating both models, a mitochondrial Ca^2+^ transient was described by Lu et al. (2013), which has some key differences when compared to its cytosolic counterpart. As the firing rate of the AP increases, a decrease in amplitude maintains a more stable concentration throughout the whole AP by slowly increasing diastolic Ca^2+^ concentrations, although a faster decline was also noted compared to baseline after stimulating with a catecholamine analog (Lu et al., 2013). It is estimated that this happens because mitochondrial structures pump Ca^2+^ into the cytosol more slowly, primarily the mitochondrial Na^+^/Ca^2+^ exchanger (mNCX), as opposed to the combined pumping force of SERCA in the SR and the sarcolemmal NCX. While it has been calculated that the percentage of cellular Ca^2+^ taken up by mitochondria is modest (Bassani et al., 1994), it can have significant effects on excitation-contraction-energetics coupling (ECEC). The main reason is that by increasing mitochondrial Ca^2+^ concentration, dehydrogenases from the Krebs cycle change to a more active form. In turn, these dehydrogenases produce more high-energy products (NADH^+^ and FADH_2_) for the electron transport chain (ETC) to use as substrates and generate the mitochondrial membrane potential (ΔΨm) and subsequent ATP synthesis under energy-demanding states, such as when adrenergic stimulation takes place (Fernandez-Sada et al., 2014; Kwong et al., 2015). This ATP is then transported to the cytosol, where it is used by the sarcomere to relax its myofibrils and, equally necessary, used by a wide range of pumps to maintain ion balance. One of these pumps is SERCA, which returns most Ca^2+^ ions, about 70% (Bassani et al., 1994), into the SR after the AP finishes and reestablishes the basal Ca^2+^ concentration. This is just one example of how mitochondria can communicate with the SR under demanding and stressful conditions, according to ECEC.

## Mitochondrial Ca^2+^ Signaling and Cardiac Arrhythmia

High cytosolic Ca^2+^ concentrations trigger mitochondrial Ca^2+^ transport by the mCU. Under homeostatic conditions, this process is finite, and Ca^2+^ can slowly be transported back into the cytosol by the mNCX. However, if Ca^2+^ cannot be extruded out of the mitochondria before more Ca^2+^ enters the mCU, then Ca^2+^ overload ensues. High mitochondrial Ca^2+^ concentrations cause higher ROS production, loss of mitochondrial membrane integrity with subsequent ΔΨm loss, and mPTP opening (Kazak et al., 2017). This last event is an initial step in the signaling cascade for the mitochondrial-mediated apoptosis pathway, causing further alterations in heart rhythmicity and organization at a cellular level. Whether SR or mitochondrial dysfunction causes the initial Ca^2+^ overload is debatable. Nonetheless, it is a safe bet to argue that this process may be self-maintained. Based on murine models of HF and AF associated with RyR2, as well as atrial samples from patients with chronic AF, the Marks group has reported that increased RyR2-mediated leakage is a potential mechanism for augmented mitochondrial Ca^2+^ content and ROS production. Post-translational analysis has revealed increased RyR2 oxidation and dissociation of the RyR2 inhibitory protein, calstabin2 or FKBP, which may further exacerbate RyR2 leakage. Thus, a feedback loop mechanism in Ca^2+^ signaling between the SR and mitochondria promotes intracellular Ca^2+^ mishandling. The SR-mitochondria interconnection in Ca^2+^ fluxes may offer the possibility of targeting mitochondrial Ca^2+^ overload to ameliorate RyR2-mediated leakage (Santulli et al., 2015; Xie et al., 2015). The importance of mitochondria in this vicious cycle is exemplified in a recent study conducted by Hamilton et al. (2020), who demonstrated that mitochondrial ROS targeting ameliorates RyR2 leakiness in a mouse model of catecholaminergic polymorphic ventricular tachycardia (CPVT). As part of the Ca^2+^-handling proteins, mitochondrial membrane components *per se* are susceptible to redox modulation, which contributes to altered Ca^2+^ homeostasis in the myocyte. This is the case for the mCU, whose oxidation at cysteine 97 activates the channel, leading to mitochondrial Ca^2+^ overload and ROS production (Dong et al., 2017). The impact of altered mitochondrial Ca^2+^ in membrane excitability and arrhythmia has also been investigated. Myocytes from non-ischemic HF showed increased mitochondrial Ca^2+^ transients, enhanced LTCC currents, AP prolongation, and the appearance of EADs (Xie et al., 2018). Cardiac activity monitored by telemetry has demonstrated QT prolongation and a high incidence of ventricular arrhythmia following the application of isoproterenol (ISO), a catecholamine analog.

A potential causal role of mitochondrial Ca^2+^ signaling in arrhythmia is illustrated by genetically and pharmacologically blocking the mCU. We previously reported that selective mCU inhibition has the therapeutic potential to prevent catecholamine-induced toxicity, as observed in HF (Fernandez-Sada et al., 2014). In this regard, using mCU transgenic mice, two independent groups confirmed our findings of the mCU’s critical role in matching the metabolic output during the adrenergic response (Kwong et al., 2015; Wu et al., 2015). Blocking the mCU with ruthenium red (RuR) or Ru 360 min before the induction of ischemia-reperfusion (I/R) injury prevents the appearance of ventricular arrhythmia in rat models (Garcia-Rivas Gde et al., 2006; Menezes-Rodrigues et al., 2020). In contrast, other studies have reported that enhancing Ca^2+^ fluxes between the SR and mitochondria using mitochondrial Ca^2+^ uptake enhancers is beneficial in preventing the appearance of ventricular arrhythmia associated with RyR2 dysfunction (Schweitzer et al., 2017). In atrial myocytes, the Blatter group has reported that inhibiting the mCU with Ru360 aggravates the Ca^2+^ alternans ratio and decreases the alternans’ pacing threshold. These arrhythmogenic effects are restored when myocytes are treated with spermine, suggesting that mitochondrial Ca^2+^ uptake could be a potential candidate for AF treatment (Oropeza-Almazan and Blatter, 2020). Thus, the effect of blocking mitochondrial Ca^2+^ uptake, either protecting from or promoting arrhythmia, may be dependent on the cardiac condition associated with Ca^2+^ overload. For example, in I/R injury, where a massive and acute Ca^2+^ imbalance occurs, closing the mCU prevents mitochondrial damage, whereas stimulated Ca^2+^ transport in AF could ameliorate the emerging Ca^2+^-triggered arrhythmia by dissipating Ca^2+^ waves and lowering diastolic Ca^2+^ levels.

## Mitochondrial Energetic Debacle in Cardiac Arrhythmia

About 90% of the heart’s ATP is produced through oxidative phosphorylation in mitochondria (Harris and Das, 1991). Given that the heart beats continuously, cardiac muscle contraction is an extremely ATP-demanding process. Furthermore, mitochondria constitute about 25% of the volume of human cardiomyocytes, even more so in rats and mice (∼30%) (Schaper et al., 1985), which reflects the organelle’s relevance in cardiac tissue. Rising cytosolic Ca^2+^ levels during the AP stimulates Ca^2+^ transport into the mitochondrial matrix. This rising intramitochondrial Ca^2+^ concentration activates dehydrogenases involved in the Krebs cycle, which, in turn, supplies the ETC with NADH^+^ and FADH_2_ to meet the cell’s energetic demand (McCormack and Denton, 1990; Glancy and Balaban, 2012; Fernandez-Sada et al., 2014). In this sense, in cardiac cells, the Ca^2+^ signal is coupled with the energy demand through a process called ECEC. This process links Ca^2+^ handling and the contraction of the cardiomyocyte with relaxation and ATP production in mitochondria (Maack and O’Rourke, 2007). Energetic depletion can affect highly dependent ion channels and transporters that participate in proper intracellular Ca^2+^ handling. A prominent example of such a protein is SERCA, which needs ATP to transport Ca^2+^ into the SR during myocardium relaxation (Bers, 2002). Impairing SERCA’s functioning reduces SR Ca^2+^ content and increases the cytosol’s diastolic Ca^2+^ concentration. Another example of an ATP-driven protein function is sarcKATP, a potassium ion channel in the sarcoplasmic membrane that opens upon a low ATP/ADP ratio. High ATP/ADP ratios usually inhibit this channel, which limits the reactivation of inward currents by prolonging the refractory period and avoiding functional re-entry. The O’Rourke lab recently proposed that sarcKATP channels are the means by which changes in the ΔΨm cause altered cellular membrane excitability. In their work, metabolic dysfunction induced by the mitochondrial uncoupler carbonyl cyanide-p-trifluoromethoxyphenylhydrazone (FCCP) increased IKATP and shortened the AP and refractory period, leading to re-entry into myocyte monolayers. The proarrhythmogenic effects of FCCP are attenuated by the IKATP inhibitor glibenclamide (Zhou et al., 2014). Depleting mitochondrial ATP production may impact membrane excitability by promoting T-wave alternans. In the electrocardiogram (ECG), T-wave alternans are linked to ventricular arrhythmia and constitute a prominent arrhythmogenic mechanism in the settings of myocardial infarction and ischemia, conditions characterized by the depletion of mitochondrial ATP production. T-wave alternans reflect beat-to-beat variation in the action potential duration (APD) at a cellular level, which are known as APD alternans, and numerous studies have reported that they are coupled to intracellular Ca^2+^ release variations (Ca^2+^ transient alternans). In an isolated rabbit heart, FCCP perfusion caused a higher incidence of APD and Ca^2+^ transient alternans, increased interventricular heterogeneity, impaired cardiac conduction velocity, and promoted ventricular arrhythmias in optical mapping studies. In this study, APD and Ca^2+^ alternans were also induced under ischemic conditions in rabbit heart preparation, validating the clinical relevance of blunted mitochondria energetics in cardiac arrhythmogenesis (Smith et al., 2013). In contrast, several other studies have reported that mitochondrial uncoupling proteins (UCPs) confer protection against cardiac arrhythmias in conditions of I/R. Subjecting cardiac UCP3 knockout (UCP3^–/–^) mice to I/R injury produced twofold larger infarcts, a high propensity for ventricular tachycardia, and excessive ROS generation when compared to WT mice. Pretreatment with FCCP prior to I/R injury relieved cardiac stress in UCP3^–/–^ hearts and improved ventricular functioning (Ozcan et al., 2013). Thus, the balance between mitochondrial energetics preservation should be a target for better cardiac arrhythmia protection in cardiac ischemia. Mitophagy, the degradation of mitochondria by autophagy, is considered a protective mechanism against the damage that mitochondria experience during I/R injury. Adenoviral expression of the uncoupling protein 2 (UCP2) in the heart promoted mitochondria mitophagy in the settings of I/R injury and offered cardiac output preservation comparable with ischemic preconditioning (Wu et al., 2019). The significance in cardiac arrhythmia was not addressed in this study, but it may be the case since another study reported that the genetic ablation of UCP2^–/–^ in mice resulted in AP and QT shortening, leading to a high propensity for ventricular arrhythmias (Larbig et al., 2017). In the setting of AF, high AP firing imposes a high demand for ATP in the atrial chambers, causing mitochondria remodeling, altered Ca^2+^ handling, and impaired atrial contraction. In atrial-like myocytes (HL-1 cells), 6–8 h of tachypacing caused dissipation of the ΔΨm and mitochondrial network, as well as the loss of mitochondrial Ca^2+^ transients. Interestingly, treatment with SS31, a compound that accelerates the formation of ATP from ADP, protected against mitochondria remodeling, impaired respiration, and Ca^2+^ homeostasis (Wiersma et al., 2019). The AMP-activated protein kinase (AMPK) is a cellular energy sensor that activates when the AMP/ATP ratio increases. Expression of a mutant (T172D) of the AMPK α1-subunit in rat ventricular myocytes resulted in the impaired open-state inactivation of Nav1.5, which led to AP prolongation and a high incidence of EADs at the cellular level. Whether this arrhythmogenic mechanism operates in conditions of high metabolic stress, as in ischemic HF and AF genetic syndrome, has not been yet determined (Harada et al., 2012). Similarly, impaired cardiac metabolism has been associated with several other cellular changes that increase the risk of arrhythmia generation. ATP, while mainly used by pumps and contractile machinery, is also used as a second messenger. ATP by itself can bind with RyR2 or Mg^2+^, a RyR2 inhibitor, and lower the Ca^2+^ threshold needed to elicit a Ca^2+^-induced-Ca^2+^ release (Fill and Copello, 2002; Meissner, 2004). Low ATP would mean RyR2 inhibition with a subsequent deficient Ca^2+^ release. Impaired metabolism also increases phosphate levels, decrease ATP levels which, inhibit sarcolemmal Na^+^/K^+^ ATPase and activate the glycolytic pathway with the conversion of pyruvate to lactic acid, lowering the cell’s pH. The low pH activates the sarcolemmal Na^+^/H^+^ exchanger, increasing the cytosolic Na^+^ concentration. Na^+^ accumulation is further aggravated by less active Na^+^/K^+^ ATPase and the translocation of connexin hemichannels to the non-junctional membrane, which turn into non-selective cation channels during ischemia or metabolic inhibition. High cytosolic Na^+^ levels thus stimulate Ca^2+^ extrusion from the mitochondrial matrix through the mNCX, further inhibiting ATP synthesis and increasing cytosolic Ca^2+^ levels by inhibiting the sarcolemmal NCX (Yang et al., 2015).

Translocating connexin hemichannels to a non-junctional membrane, transforming them into non-selective channels, can also initiate other signaling pathways that promote arrhythmias (Kim et al., 2019). Because subsarcolemmal mitochondria also express connexin 43 in their IMM (Boengler et al., 2009), their proximity to the sarcolemma could enable the transport of messengers, such as ATP and Ca^2+^, between compartments, which could increase the risk of cellular and mitochondrial Ca^2+^ overload and arrhythmias. Another channel with properties similar to connexin is pannexin 1 (Panx1). This channel is activated by cytoplasmic Ca^2+^ (Locovei et al., 2006) and ROS (Zhang et al., 2008) and has been reported to release ATP into the extracellular matrix upon activation in fibrotic processes (Dolmatova et al., 2012). ATP released into the medium by these channels activates P2X and P2Y receptors, or it can be rapidly converted to adenosine in the extracellular matrix, binding to its G-coupled receptor, A2AR, and inducing TGF-β1 expression (Shaikh et al., 2016; Zhang J. et al., 2017). This cytokine then stimulates collagen deposition and fibrosis of the cardiac tissue (Cerrone et al., 2018), which is known to act as a physical barrier to electrical signaling conduction, facilitating re-entry and arrhythmia propagation.

## Mitochondrial Redox Imbalance and Cardiac Arrhythmia

In mitochondria, ROS is produced when electrons flowing through the ETC react prematurely with oxygen before reaching complex IV to form radical superoxide. These unstable molecules can then transform into hydrogen peroxide by mitochondrial superoxide dismutase or into hydroxyl radicals if they encounter a metal with which to react (Alfadda and Sallam, 2012). These byproducts of the ETC, while having a relatively short half-life, can diffuse to nearby cellular compartments and have various effects on a wide range of proteins. In the mitochondria-SR microdomain, proteins, such as the RyR2 and SERCA, are possible sites of protein oxidation, as well as other proteins implicated in the AP propagation that also reside within or near the dyadic cleft, all of which can affect cellular Ca^2+^ handling.

The RyR2 contains at least 21 free cysteine residues per monomer deemed to be susceptible to oxidation (Nikolaienko et al., 2018). Anzai et al. (1998) reported that hydroxyl radicals increased the opening probability of RyR2 channels reconstituted in lipid bilayers, an effect that was reverted by the SH-reducing agent dithiothreitol (Boraso and Williams, 1994; Anzai et al., 1998). At the cellular level, exposing cardiac myocytes to ROS increased the RyR2-mediated leak in permeabilized isolated ventricular myocytes, further supporting an up-regulatory effect of ROS (Terentyev et al., 2008). In another study, the β-adrenergic stimulation of myocytes promoted ROS production, oxidized RyR2, and, along with the RyR2 phosphorylation, enhanced the appearance of Ca^2+^ waves. MitoTEMPO treatment ameliorated the incidence of Ca^2+^ waves, indicating that mitochondria play a role in the production of oxidative stress (Bovo et al., 2012).

The proper functioning of the RyR2 is assisted by associated proteins, including calstabin and the FKBP12.5 factor. This factor exerts intrinsic negative regulation of the RyR2 and disrupts the RyR2-FKBP interaction, resulting in SR Ca^2+^ leakage and atrial and ventricular arrhythmias (Wehrens et al., 2003; Sood et al., 2008). The RyR2-FKBP association is redox-regulated (Zissimopoulos et al., 2007). As mentioned previously, highly oxidized levels of RyR2 were associated with increased SR Ca^2+^ leakage and an increased propensity for AF in a mouse model of CPVT. RyR2 oxidation was correlated with FKBP depletion from the RyR2, providing the molecular basis for atrial arrhythmia. Notably, the pharmacological stabilization of RyR2-FKBP binding prevented diastolic spontaneous Ca^2+^ releases in isolated atrial myocytes and decreased the propensity for AF *in vivo* (Shan et al., 2012). Disruption of the RyR2-FKBP interaction by oxidative stress has also been identified as the mechanism by which palmitoyl-carnitine (PC) promotes RyR2 leakage and, subsequently, cardiac arrhythmia (Roussel et al., 2015). In this study, the measurement of ROS production by MitoSox showed increased mitochondrial superoxide amounts in myocytes pre-exposed to PC, suggesting that mitochondrial ROS reduces RyR2-FKBP stability.

Oxidative stress also affects the RyR2 channel’s activity by activating Ca^2+^ calmodulin kinase II (CaMKII) (Erickson et al., 2008). The clinical significance of CaMKII oxidation in atrial arrhythmogenesis was first explored by Mark E. Anderson’s group. They reported an increase in oxidized CaMKII levels in atria from patients with AF, and the causality of AF was provided in a mouse model infused with the pro-oxidant hormone angiotensin II. After 3 weeks of treatment, angiotensin caused CaMKII oxidation, promoted SR Ca^2+^ leakage in isolated atrial myocytes and increased *in vivo* susceptibility to AF. The genetic inhibition of CaMKII oxidation reduced the propensity for AF, suggesting that CaMKII acts as a coupler between oxidative stress and atrial arrhythmia (Purohit et al., 2013). In line with these findings, Zhang et al. (2014) reported that the overexpression of NADPH oxidase 4 (NOX4) in zebrafish embryos resulted in increased superoxide production, CaMKII activation, and cardiac arrhythmia. Interestingly, MitoTEMPO treatment prevented NOX4-induced H_2_O_2_ production, indicating that mitochondrial oxidative stress plays a role in cardiac arrhythmia.

The SERCA pump plays a critical role in myocyte relaxation by promoting the recapture of Ca^2+^ by the SR. This pump is also subjected to oxidative regulation. Cys674 is identified as a critical target for oxidative modification, including oxidation (Qin et al., 2013) and sulfonylation (Hobai et al., 2013). The exposure of an SR membrane preparation to H_2_O_2_ demonstrated that ROS slows down the recapture of Ca^2+^, which is consistent with an inhibitory effect (Qin et al., 2013). Loss of SERCA function caused by oxidative stress is, in part, responsible for diastolic dysfunction in aging (Herraiz-Martínez et al., 2015), inherited (Robinson et al., 2018) or acquired (Beuckelmann and Erdmann, 1992) cardiac conditions, and metabolic dysregulation (Bugger and Abel, 2014). Suppressed SERCA pump activity prolongs the Ca^2+^ spark duration life (O’Neill et al., 2004), increasing the chances of reaching the NCX and inducing triggered activity. The role of SERCA oxidation in cardiac arrhythmia in the setting of mitochondrial dysfunction deserves further exploration. As part of the SR Ca^2+^-handling proteins, ROS is implicated in the development of cardiac substrates and triggers by causing the direct oxidation of membrane ion channels and structural elements or by indirectly targeting signaling pathways that modulate their function and expression. These topics have been extensively reviewed, and we would like to refer readers to excellent reviews in the area (Zima and Blatter, 2006; Takimoto and Kass, 2007; Jeong et al., 2012; Rutledge and Dudley, 2013).

Aging is a complex multi-factorial process characterized by a progressive decline in the organ function. Mitochondrial dysfunction also increases with aging and the resulting accumulation of ROS may contribute to the aging process by gradually damaging cellular components, a theory known as “the free radical theory of aging” proposed by Harman (1956). Aging is a primary risk factor for AF, the most common arrhythmia in the elderly population. AF may be the result of the accumulation of damaged biomolecules by mitochondrial oxidative stress over the years being the mitochondrial DNA (mtDNA) one of the exposed targets. Analysis of the mtDNA integrity in atrial samples from patients with persistent AF revealed deletion of the 4977 bp, as well as a higher mitochondrial content of 8-OHdG compared with control patients, suggesting DNA damage by oxidative stress (Lin et al., 2003). A connection between oxidative stress and the pathophysiology of persistent AF may also have a genetic basis which is exemplified in the study conducted by Kim et al. (2003) who compared the transcriptional profile between control patients and patients with chronic AF using cDNA microarrays. Among those upregulated genes in atrial samples from AF patients, five were encoding enzymatic systems associated with ROS production.

Pulmonary vein (PV) dysregulation is involved in the initiation of AF and a study demonstrated that the reduction of the atrial arrhythmia burden in patients undergoing isolation of PVs correlated with significantly lower plasma levels of oxidative markers (advanced glycation end-products and thiobarbituric-acid reacting substances) 9 months after the intervention (Böhm et al., 2016). From the experimental side, Wongcharoen et al. (2007) reported that PVs isolated from aged rabbits showed more depolarized resting membrane potentials and larger amplitude of EADs compared young PV preparations. Whether the high cellular arrhythmogenicity in aged PVs is linked to oxidative stress remains to be elucidated. Atrial remodeling, which contributes to the progression of permanent AF, also increases with aging and animal studies support a causal role for oxidative stress. In this regard, pioglitazone, an activator of the peroxisome-activated receptor gamma (PPAR-γ), decreased atrial fibrosis, NADPH oxidase subunits p22phox and gp91phox expression and the duration of pacing-induced AF in aged rats (Xu et al., 2012).

Aging regulates mitochondrial Ca^2+^ dependent processes including apoptosis and energetics by directly affecting mitochondrial components or their spatial relationship to the SR. For instance, the pharmacological inhibition of mPTP is reduced in hearts from senescent rats compared to younger animals when subjected to ischemia/reperfusion injury (Zhu et al., 2010). On the other hand, decreased mitochondrial Ca^2+^ uptake occurs in old isolated cardiomyocytes which correlated with lower mitochondrial NAD(P)H/NAD(P^+^) ratio in response to electrical pacing. Interestingly, immunolabelling of permeabilized myocytes with anti-RyR and anti-VDAC revealed an age-dependent reduction of the fraction of the RyR2 overlapping with VDAC, whereas quantitative redox proteomics showed augmented oxidation of VDAC proteins. Thus, oxidation of mitochondrial membrane components may contribute to the disruption of the spatial proximity between mitochondria and the SR, which may cause defective Ca^2+^-dependent mitochondrial energetics (Fernandez-Sanz et al., 2014). Which in turn others have appointed this increase in mitochondrial ROS to dysfunctional mitochondria which oxidize the RyR2, developing an increased risk of arrhythmia (Cooper et al., 2013). This increased arrhythmia risk was reduced by MitoTEMPO (Murphy et al., 2019).

## Mitophagy in Cardiac Arrhythmia

**FIGURE 2:**
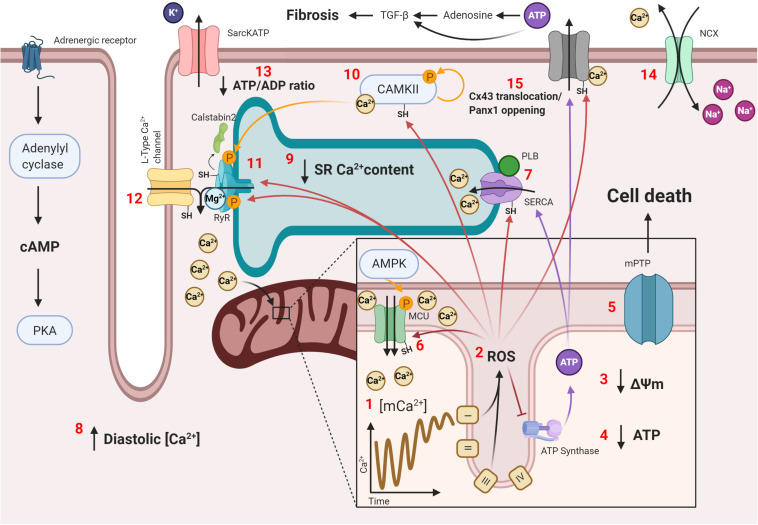
Mitochondria-SR-T tubule communication in arrhythmogenesis. Conditions that favor Ca^2+^ overload, such as chronic adrenergic stimulation, cause the following: **(1)** increased Ca^2+^ transport through the mCU to the mitochondrial matrix, shortening mitochondrial Ca^2+^ transients’ amplitude and stabilizing the mitochondrial Ca^2+^ concentration at a high level, which causes mitochondrial Ca^2+^ overload; **(2)** increased ROS production by mitochondrial structures, such as the ETC, with subsequent **(3)** loss of ΔΨm and **(4)** decreased ATP production; **(5)** opening of the mPTP, which initializes the cell death signal; **(6)** increased mCU activity by oxidation and, possibly, AMPK phosphorylation following a decline in the ATP concentration, which increases Ca^2+^ transport and ROS production; **(7)** decreased SERCA activity by oxidation and decreased PLB phosphorylation, which **(8)** increases the diastolic Ca^2+^ level and **(9)** decreases SR Ca^2+^ content, reducing contractility; **(10)** increased CAMKII activity by Ca^2+^ activation, oxidation, and autophosphorylation; **(11)** sensibilization of the RyR2 to luminal Ca^2+^ content with a reduced refractory state by oxidation and CAMKII hyperphosphorylation, with a subsequent unbinding of its modulating protein, calstabin2, and lower sensitivity to cytosolic Ca^2+^ by Mg^2+^ binding; **(12)** oxidation of LTCCs, which increases the amount of Ca^2+^ entering the cardiomyocyte upon activation; **(13)** opening of sarcKATP channels due to a reduced ATP/ADP ratio, which decreases the cardiomyocyte’s bathmotropism and dromotropism; **(14)** increased activity of the NCX, which slowly depolarizes the cardiomyocyte and increases the risk of an unsolicited AP; **(15)** connexin 43 (Cx43) translocation in certain cardiac pathologies or pannexin 1 (Panx1) opening with Ca^2+^ or ROS transports ATP into the extracellular matrix, where it is transformed into adenosine, and both ATP and adenosine cause TGF-β1 expression and fibrosis. All these changes combined produce, at the cellular level, Ca^2+^ mishandling with subsequent low contractility and spontaneous contraction that can propagate to neighboring cardiomyocytes and, at the tissular level, patches of slow conduction by reduced dromotropism or collagen deposition caused by cardiomyocyte cell death and fibrosis, which enable possible re-entry zones for sustained arrhythmias.

Since proper mitochondrial functionality is needed to prevent potentially damaging environments that predispose cardiac tissue to arrhythmias, defective remodeling of these organelles is also a potential mechanism for mitochondrial dysfunction with subsequent ROS generation and Ca^2+^ mishandling. Such is the case in the aging heart, were defective mitophagy has been described to ensue. This has been shown to cause accumulation of dysfunctional mitochondria and spontaneous Ca^2+^ release under adrenergic stimulation, which were prevented upon treatment with Torin1, an autophagy enhancer (Murphy et al., 2019). Another example is the SPRED2 deficiency murine model, an intracellular inhibitor of the ERK-MAPK pathway. SPRED2^–/–^ mice have been described to develop cardiomyocyte hypertrophy, fibrosis, impaired excitability, and arrhythmias, which were associated with impaired mitophagy and the accumulation of defective mitochondria, along with increased ROS production, caspase 3 activity, and apoptosis, as this pathway seems to inhibit normal autophagy signaling. Selumetinib, a MAPK signaling inhibitor, restored autophagic flux *in vivo*, although no effects on arrhythmias were tested (Ullrich et al., 2019). Nonetheless, this proves the importance of proper mitochondrial remodeling to prevent damage accumulation and provides yet another possible therapeutic target, mitochondrial dynamics. The term mitochondrial dynamics refers to the changes by which individual mitochondria go through the process of fusion and fission within the cell to isolate damaged mitochondria or to better couple the generation of ΔΨm to ATP synthesis, respectively. This is important in certain cardiac pathologies, such as HF. Pressure-overload HF murine models have been described to have an mitophagic blockade, which causes damaged mitochondria to accumulate and mitochondrial dysfunction to ensue (Yu et al., 2018). Regarding the different states of the mitochondrial network, fused mitochondria predomination has been described to be beneficial for cardiac function (Wai et al., 2015; Qin et al., 2020), as they are better at ATP synthesis and have lower ROS generation. Following this train of thought, inhibiting fission, promoting fusion, or maintaining an adequate mitophagy profile could potentially protect cardiac electrical signaling and organization. A summary with the presented mechanisms of arrhythmias are shown in Figure 2.

## Novel Targets in SR-Mitochondria Interconnection as Potential Anti-Arrhythmic Drugs

The development of new therapeutics could break the vicious cycle of Ca^2+^ overload, mitochondrial dysfunction, and ROS generation to substantially reduce the incidence of sudden cardiac death and disease progression, especially in high-risk patients, such as those with HF. Fortunately, there are a few candidates that may prove useful in doing so. A summary of such compounds can be reviewed in Table 1.

**TABLE 1:** Summary of therapeutic strategies used to prevent arrhythmogenic events and their main findings.

Therapeutic target	Compound	Mechanism of action	Models used	Main findings	References
mCU	Ru_36__0_	Prevents mitochondrial Ca^2+^ overload by inhibiting mitochondrial Ca^2+^ transport	Pacing-induced VF model; I/R murine model; NI-HF murine model; pressure overload HF model	Reverted VF into VT; prevented arrhythmias upon reperfusion; prevented DADs in isolated cardiomyocytes; and VF when challenged with ISO; better mitophagy profile	Kimura et al., 2005; Garcia-Rivas Gde et al., 2006; Hamilton et al., 2018; Xie et al., 2018; Yu et al., 2018
	Efsevin	VDAC2 enhancer, increases Ca^2+^ transport into mitochondria	Zebra fish cardiac tissue	Prevented spark propagation into Ca^2+^ waves	Shimizu et al., 2015; Schweitzer et al., 2017
	Kaempferol	mCU enhancer, increases Ca^2+^ transport into mitochondrial matrix	CPVT murine model and iPSC-derived human cardiomyocytes	Prevented diastolic Ca^2+^ waves and spontaneous AP in both models as well as VT *in vivo*	Schweitzer et al., 2017
	Spermine	mCU enhancer, increases Ca^2+^ transport into mitochondrial matrix	Tachypacing protocol in atrial rabbit cardiomyocytes	Lowered Ca^2+^ transient alternans	Oropeza-Almazan and Blatter, 2020
	CGP-37157	mNCX inhibitor, reduces Ca^2+^ transport out of the mitochondria	Porcine model of I/R	Suppressed ventricular arrhythmias, lowered J point depression and expedited ST segment resolution	Sventzouri et al., 2018
	Cariporide	N^+^/H^+^ exchanger inhibitor, potential regulator of mitochondria uptake	I/R model; isolated cardiomyocytes with ouabain poisoning; cardiomyocytes with angiotensin II and endothelin 1 administration	Prevented reperfusion arrhythmia, preserved metabolic status; prevented Ca^2+^ overload, delayed mPTP opening and hyper contracture; reduced ROS production and Ca^2+^ induced swelling	Sugiyama et al., 1999; Toda et al., 2007; Garciarena et al., 2008
mROS	Glutathione	ROS scavenger to prevent ROS damage	I/R in isolated rat hearts	Reduced reperfusion arrhythmias	Woodward and Zakaria, 1985
	Ascorbic acid	ROS scavenger to prevent ROS damage	I/R in isolated rat hearts	Reduced reperfusion arrhythmias; reduced lipid peroxidation, preserved mitochondria respiratory function and improved survival	Woodward and Zakaria, 1985; Tsai et al., 2011
	N- acetylcysteine	Glutathione precursor	I/R in isolated rat and dog hearts; murine model of fatty acid accumulation	Reduced reperfusion arrhythmias, reduced infarct size, improved mechanical function recovery; Preserved ΔΨm and Ca2+ transient amplitude, reduced Ca2+ sparks and prevented VT	Qiu et al., 1990; Sochman et al., 1990; Roussel et al., 2015
	TEMPOL	ROS scavenger to prevent ROS damage	I/R in rat model	Reduced arrhythmias, lipid peroxidation, preserved mitochondria respiratory function and improved survival	Tsai et al., 2011
	MitoTEMPO	ROS scavenger to prevent ROS damage	ISO-exposed isolated rabbit cardiomyocytes; CPVT murine model; HF guinea pig model; human end-stage HF cardiomyocytes	Partially prevented SR Ca2+ leak, Ca2+ waves, and ROS production; reduced ROS generation with subsequent RyR2 oxidation and leakage; prevented sudden cardiac death, ventricular arrhythmias and remodeling; reduced RyR2 hyperactivity and Ca2+ waves	Bovo et al., 2012; Ni et al., 2016; Dey et al., 2018; Dries et al., 2018; Hamilton et al., 2020
	SS-31 and SS-20	Most likely ROS scavenger and ROS production inhibitor	I/R model	Reduced infarct size, severity, and duration of arrhythmias and lipid peroxidation	Cho et al., 2007
	Quercetin / quercetin-filled liposomes	ROS scavenger to prevent ROS damage	I/R murine and dog model	Reduced arrhythmias and platelet aggregation; prevented peroxynitrite-induced arrhythmias	Xiao et al., 1993; Soloviev et al., 2002; Lozano et al., 2019
	Resveratrol	ROS scavenger, sirtuin activator	I/R murine model; cardiomyocytes from human-induced pluripotent stem cells from patients with very long-chain acyl-CoA dehydrogenase deficiency	Reduced reperfusion arrhythmias and mortality, decreased LDH and increased NO levels; reduced DADs and cytosolic Ca2+ levels as well as normalizing APD by decreasing fatty acid oxidation intermediates	Hung et al., 2000; Knottnerus et al., 2020
	DTT	Restores reduced thiol groups in oxidized proteins	Isolated atrial cardiomyocytes exposed to TNF-α	Decreased transient amplitude, spark frequency and duration, and ROS production	Zuo et al., 2018
mPTP	CsA	Inhibits mPTP opening	I/R murine model; chronic AV block canine model; VF murine model; rabbit I/R model	Reduced arrhythmias, cardiac edema and cardiomyocyte death; reduced hypertrophy and arrhythmia susceptibility, no changes in electrical remodeling; no protection found; CsA offered no protection	Arteaga et al., 1992; Schreiner et al., 2004; Brown et al., 2008; Ayoub et al., 2017
	Bongkrekic acid	Inhibits ANT, reducing Ca2+ overload and ROS production	Murine model of fatty acid accumulation	Preserved ΔΨm and Ca2+ transient amplitude, reduced ROS production, RyR2 oxidation, Ca2+ sparks and prevented VT	Roussel et al., 2015
	Octylguanidine	Inhibits mPTP opening	I/R murine model; hyperthyroid I/R murine model	Prevented arrhythmias and edema, preserved blood pressure, and mitochondrial function; prevented arrhythmias and preserved hemodynamic parameters, mitochondrial function, and reduced inflammatory cytokines	Parra et al., 2005; Pavón et al., 2009
	Citicoline	Inhibits mPTP opening	I/R	Prevented arrhythmia and pressure drop during reperfusion, maintained ΔΨm and reduced ROS damage	Hernández-Esquivel et al., 2014
	4-ClDzp	Ligand to TPSO, a mPTP regulator	Rabbit I/R model; guinea pig I/R	Preserved cardiac function; preserved ΔΨm, reduced AP shortening and prevented reperfusion arrhythmias	Akar et al., 2005; Brown et al., 2008
CAMKII	KN-93	CAMKII inhibitor	Genetic murine models: Gain-of-function RyR2, CAMKII overexpression, constitutively active CAN, CPVT Diabetic hyperglycemia murine model Human cardiomyocytes from diabetic HF patients Cardiomyocytes derived from induced pluripotent stem cells from CPVT patients Trabeculae from HF patients	Prevented AF after fast pacing; prevented arrhythmias after ISO administration; decreased premature contractions and sparks; improved contractile function	Khoo et al., 2006; Chelu et al., 2009; Sag et al., 2009; Sossalla et al., 2010; Liu et al., 2011; Di et al., 2013; Erickson et al., 2013
	AIP	CAMKII inhibitor	CAMKII overexpressing murine model; trabeculae from HF patients; impaired glucose tolerance murine model	Prevented afterdepolarization in isolated cardiomyocyte; improved contractile function; prevented arrhythmias and arrhythmogenic cellular events	Sag et al., 2009; Sossalla et al., 2010; Federico et al., 2019
RyR2	Dantrolene	RyR2 stabilizer	CPVT KI murine model *Ex vivo* rabbit heart Long QT syndrome model Atrial infarct sheep model Cardiac arrest pig model Isolated cardiomyocytes from AF or endstage HF patients Cardiomyocytes from induced pluripotent stem cells from CPVT patients Clinical trial with CPVT patients	Prevented VT and decreased spark frequency; reduction of EADs and VT frequency; prevented AF; same efficacy as amiodarone to regain spontaneous circulation and hemodynamic stability in cardiac arrest; reduced spark frequency, Ca2+ waves, Ca2+ leak and spontaneous transients; reduced premature ventricular beats	Kobayashi et al., 2010; Penttinen et al., 2015; Hartmann et al., 2017; Wiesmann et al., 2017; Avula et al., 2018; Frommeyer et al., 2018
Calstabin2	JTV519	Increases affinity of calstabin2 to the RyR2	HF dog model KD calstabin2 murine model HEK293 with CPVT Ry mutations *in vitro* model 3D engineered heart tissue with optical activated ion channel model	Abolished abnormal RyR2 gating; prevented VT and sudden cardiac death	Kohno et al., 2003; Lehnart et al., 2004a; Wehrens et al., 2004; Lemme et al., 2019
	S107	Increases affinity of calstabin2 to the RyR2	CPVT murine models DMD murine model Cardiomyocytes derived from induced pluripotent stem cells from CPVT patients CPVT analogue nematode model	Prevented arrhythmias and seizures; reduced Ca2+ leak; prevented DADs	Lehnart et al., 2008; Fauconnier et al., 2010; Shan et al., 2012; Sasaki et al., 2016; Fischer et al., 2017
Mitochondrial dynamics	Mdivi-1	Mitochondrial fission inhibitor	I/R murine model	Reduced arrhythmias and preserved hemodynamic parameters	Maneechote et al., 2018
	M1	Mitochondrial fusion promoter	I/R murine model	Reduced arrhythmias and preserved hemodynamic parameters	Maneechote et al., 2019
mitoKATP	4CPI	H2S pro-drug (a mitoKATP agonist)	I/R model	Reduced tissue injury, ROS production, prevent reperfusion arrhythmias and improved recovery	Testai et al., 2016
	Compound A	mitoKATP agonist	I/R model	Reduce reperfusion arrhythmias, better results than diazoxide, no added effect with sarcKATP blocker	Gonca et al., 2016

### Regulation of Mitochondrial Ca^2+^ Transport

Considering that the mCU seems to be the central piece in the positive feedback cycle, mCU inhibition may be efficient in stopping the cycle and maintaining mitochondrial integrity. Ru_360_ is a potent mCU inhibitor. It is an oxygen-bridged small molecule with a dinuclear ruthenium center complexed with amine groups. This molecule was first fully described in 1998 and synthesized from a familiar component, RuR. RuR was first used as the mCU inhibitor to study mitochondrial Ca^2+^ dynamics but was found to be non-specific for the mCU, as it has various effects on other cellular components. Ru_360_ was then described as being more potent (IC50 of 0.184 vs. 6.85 nM for RuR) and having a much greater affinity for the mCU, with a Kd of about 0.34 nM. Doses of up to 10 μM had no effect on SERCA, the RyR2, NCX, LTCCs, or myofibrils (Matlib et al., 1998; García-Rivas et al., 2005), which demonstrates its high specificity for the mCU and safety. In this sense, Ru_360_ treatment partially inhibited the mCU, maintaining proper mechanical performance of the heart and mitochondrial matrix free-Ca^2+^ concentrations at basal levels, despite high concentrations of cytosolic Ca^2+^ after ischemia/reperfusion (I/R) injury (García-Rivas et al., 2005). In an open-chest rat model of I/R, a bolus of Ru_360_ given before ischemia significantly prevented the occurrence of ventricular fibrillation (VF) and hemodynamic dysfunction. Ru_360_ administration was associated with protecting mitochondria from depolarization by mPTP opening and thus preserved mitochondrial functioning (Garcia-Rivas Gde et al., 2006). Ru_360_ infusion also reverted the progression of pacing-induced sustained VF into ventricular tachycardia, a less severe form of arrhythmia, in an isolated heart model. This was associated with a reduction in the APD, refractory period, and slope of the APD restitution curves (Kimura et al., 2005).

Since then, Ru_360_ has been used to research the effects of inhibiting mitochondrial Ca^2+^ transport on various models of cardiac injury. It has already proved its efficacy in preventing EADs in ventricular myocytes in a non-ischemic HF model (Xie et al., 2018). It has also been shown to reduce VF in mice with thoracic aorta banding when presented with a catecholamine challenge. It is worth mentioning that inhibiting mitochondrial Ca^2+^ transport was related to less oxidized RyR2 in isolated cardiomyocytes, as opposed to other drugs that increase mitochondrial Ca^2+^ content, such as kaempferol, the mCU agonist, and CGP-37157, the mNCX inhibitor, which enhances RyR2 oxidation. Isolated cardiomyocytes also presented with improved Ca^2+^ homeostasis and less spontaneous Ca^2+^ waves (Hamilton et al., 2018). This further proves that mitochondrial Ca^2+^ overload and ROS generation can affect SR components and should be taken into consideration more fully. Finally, mitochondrial Ca^2+^ transport inhibition was described as useful in maintaining proper ECG signaling in a murine pressure-overload HF model by enhancing mitochondrial autophagy (Yu et al., 2018). It has been previously described that mitochondrial dynamics (i.e., fusion, fission, and mitophagy) are impaired in a wide range of cardiovascular diseases and could be another potential therapeutic target (Vásquez-Trincado et al., 2016). Unsurprisingly, mitophagy was found to be impaired in the HF model and inhibiting mitochondrial Ca^2+^ transport seemed to prevent the accumulation of dysfunctional mitochondria, although no clear explanation was given for why this happened. It appears that there is much more to be described regarding the affected mitochondrial signaling mechanisms when mitochondrial Ca^2+^ overload ensues.

Inhibiting mitochondrial Ca^2+^ transport seems to be a plausible therapeutic strategy to prevent arrhythmias. Nonetheless, there is evidence indicating that the opposite approach would be more effective. It has been observed that efsevin, a VDAC2 enhancer, allows better Ca^2+^ transport from the SR to mitochondria by interacting directly inside the channel pore (Wilting et al., 2020). This enhanced Ca^2+^ transport was observed to abolish Ca^2+^ spark propagation into Ca^2+^ waves and erratic contractions by limiting the Ca^2+^ spark’s temporal and spatial boundaries, effectively acting as a “Ca^2+^ sponge” (Shimizu et al., 2015). Similarly, in a CPVT murine model, diastolic Ca^2+^ waves and spontaneous APs in isolated cardiomyocytes were prevented by enhancing mitochondrial Ca^2+^ transport via either efsevin or the mCU enhancer, kaempferol. Ventricular tachycardia was prevented *in vivo*, and similar results were found in iPSC-derived human cardiomyocytes (Schweitzer et al., 2017). Furthermore, enhancing mitochondrial Ca^2+^ content using the mNCX inhibitor CGP-37157 suppressed ventricular arrhythmias, lowered the depression of the J point during ischemia, and expedited ST-segment resolution after reperfusion in a porcine model of I/R (Sventzouri et al., 2018). Likewise, enhancing mitochondrial Ca^2+^ transport using the mCU activator spermine in isolated rabbit atrial cardiomyocytes undergoing a tachypacing protocol to induce AF demonstrated a protective effect against Ca^2+^ transient alternans. However, inhibiting mCU Ca^2+^ transport with Ru_360_ lowered the threshold needed to achieve alternans and enhanced the severity of the alternans (Oropeza-Almazan and Blatter, 2020).

Similar to the approach of using a specific mCU inhibitor, using gene therapy to downregulate mCU expression is another feasible alternative. With the development of better protocols to direct gene therapy to specific tissues, it may be possible to knockout (KO) mCU expression using siRNA (Oropeza-Almazan et al., 2017). Transgenic mice with mCU KO in cardiac tissue were shown to have less mPTP opening than their WT counterparts and appeared to have no problems when resting and upon being put on a treadmill. These mCU KO mice could even sprint for as long as their WT counterparts, provided that they were given a 30-min “warm-up” beforehand, as they were unable to do so acutely (Kwong et al., 2015). This finding correlates with the finding of similar mitochondrial basal Ca^2+^ levels between groups, while the KO group needed more time to accumulate Ca^2+^ after stress stimulation. Cardiomyocytes from the KO group also showed less mPTP opening compared to the WT group, and the KO hearts had a smaller affected area after I/R injury. Another study using mice with mCU knockdown in cardiac tissue showed that upon being submitted to a non-ischemic HF model, they presented with reduced Na^+^/Ca^2+^ exchange currents, decreased APD, no observed EADs, and a reduced incidence of VF compared to the WT group (Xie et al., 2018). Combining these findings with the recent development of nanometric particles used for drug delivery places this type of therapy in the possible near future, as it has been recently proven that siRNA can be packaged and protected from degradation while maintaining its biological effects *in vitro* studies. Furthermore, cariporide, an N^+^/H^+^ exchange inhibitor, has proven useful in not only preventing arrhythmias in an I/R model but also preserving the metabolic status of the heart (Sugiyama et al., 1999). At the mitochondrial level, cariporide has also been shown to have mitochondrial protective properties. In isolated ventricular myocytes, cariporide prevented mitochondrial Ca^2+^ overload upon ouabain administration. No effect was seen in preventing ΔΨm reduction, although mPTP opening was delayed significantly. This translated the ouabain taking longer to cause hyper contracture, which would indicate global ATP depletion and, thus, more preserved mitochondrial functioning (Toda et al., 2007). Notably, cariporide decreased Ca^2+^-induced mitochondrial swelling, comparable to the effects of Cyclosporine A (CsA) (Garciarena et al., 2008). N^+^/H^+^ exchange inhibitors seem to be a promising therapeutic approach for cardioprotection, although they require further studies to elucidate their precise mechanism of action in regulating mitochondrial Ca^2+^ handling to better design a therapeutic strategy.

### Modulation of Redox Homeostasis

#### Cellular ROS Scavengers

Since increased mitochondrial ROS generation seems to be the initial insult that starts the positive feedback loop, antioxidants could be useful in boosting the myocardium’s ROS defense pool. Since their first trials in animal models, ROS scavengers have had beneficial effects in preventing arrhythmias during I/R. Superoxide dismutase, glutathione, and ascorbic acid significantly reduced reperfusion arrhythmias in isolated rat hearts (Woodward and Zakaria, 1985). N-acetylcysteine, a precursor for glutathione synthesis, also demonstrated protection against reperfusion arrhythmias in isolated rat heart (Qiu et al., 1990) and dog models (Sochman et al., 1990) of I/R, reducing infarct size, preventing reperfusion arrhythmias, and improving the recovery of mechanical functioning during reperfusion. Another dog model of I/R was infused with superoxide dismutase. High-dose infusion was correlated with a reduction in ventricular extrasystoles, improved regional contractile force, and increased blood flow during reperfusion (Kónya et al., 1992). Other non-specific antioxidants, such as ascorbic acid and tempol, have also proven useful in preventing necrosis and dysfunction after VF and cardiopulmonary resuscitation (CPR). VF was induced in rats, and after 5 min, CPR and electrical shocks were used to induce a return to spontaneous circulation. Administering ascorbic acid or tempol at the start of CPR in a murine model of I/R was correlated with reduced myocardial necrosis and lipid peroxidation, preserved mitochondrial respiratory function, and a higher and faster rate of return of spontaneous circulation, along with better survival rates at 72 h post-CPR (Tsai et al., 2011).

Since the endoplasmic reticulum (ER) is yet another site where redox signaling is integrated, it is a potential ROS control organelle. Selenium (Se) is essential for the function of at least 25 selenoproteins, including the antioxidant glutathione peroxidase (GPx). In patients undergoing primary coronary artery bypass grafts, preoperative reduced levels of Se, GPx, and malondialdehyde correlated with high incidence of postoperative AF (McDonald et al., 2016). Moreover, low Se concentration is inversely associated with coronary heart diseases in observational studies (Flores-Mateo et al., 2006). In animal studies, administration of selenoprotein T (PSELT5) prevented the increase of cardiac ROS levels and infarct size area following I/R injury (Rocca et al., 2018). In primary culture of cardiomyocytes, low concentration of Se impaired mitochondrial function and reduced expression of Kv1.2 channel (Zhang C. et al., 2017). Although no electrophysiologic consequences were provided in this study, a reduced I_K1_ would predict AP prolongation and the facilitation of EADs. Recently, it has been described that ATF6, an ER transcription factor, is responsible for inducing antioxidative stress programs, such as catalase and ROS scavenging selenoproteins, in a setting with a high level of accumulation of misfolded proteins, such as in I/R (Jin et al., 2017). Selenoproteins were also shown to recover contractility and reduce infarct size in an isolated rat heart I/R model (Rocca et al., 2018) and treatment with dexmedetomidine, a sedative and analgesic with antioxidant properties, along with Na_2_SeO_3_, a Se supply for selenoproteins synthesis, was shown to confer cytoprotection against cellular damage in a cellular model of oxygen and glucose deprivation/reoxygenation by reducing apoptosis, LDH release, and ROS levels (Wang et al., 2020). However, the clinical application of Se should be taken with caution since it was demonstrated that the administration of this element in a patient population with acute myocardial infarction associated with HF did not prevent the appearance of ventricular arrhythmia (Tränkmann et al., 1999). Thus, it is possible that supplementation of selenium may be more beneficial as a preventive therapy. Nonetheless, this places the possibility of targeting the ER antioxidant resources as a mean to keep ROS levels down in a pathological setting with a high risk of arrhythmia.

Other antioxidants, such as quercetin and resveratrol, have been useful in preventing cellular damage and preserving mitochondrial functioning upon challenge with insults *in vitro* models, such as I/R (Lozano et al., 2019), and in reducing right-heart remodeling and dysfunction in a murine model of pulmonary arterial hypertension (Vázquez-Garza et al., 2020). Regarding arrhythmias, treatment with quercetin has proven useful in preventing platelet aggregation and arrhythmias in an I/R murine model. The protective effect is thought to be partially caused by inhibiting platelet aggregation and thus preventing microthrombi during reperfusion, which would cause heterogenous circulation when blood flow is restored, predisposing the cardiac tissue to arrhythmias (Xiao et al., 1993). A more recent study using treatment with quercetin-filled phosphatidylcholine liposomes was able to prevent peroxynitrite-induced arrhythmias in isolated murine papillary muscle and in an I/R dog model (Soloviev et al., 2002).

Resveratrol was shown to reduce lactate dehydrogenase levels, an indicator of cellular damage, as well as increase NO levels in carotid blood during I/R arrhythmias, although there was no effect on ischemia-induced arrhythmias and mortality (Hung et al., 2000). It was also observed that cardiomyocytes from human-induced pluripotent stem cells from patients with very long-chain acyl-CoA dehydrogenase deficiency presented with a shorter APD, as well as a greater incidence of DADs and higher cytosolic Ca^2+^ concentration. Resveratrol treatment reportedly abolished all these changes in one of the two patients enrolled in the study, presumably by increasing the amount of defective mitochondrial proteins, which had residual activity; however, the other patient had a more severe form of the disease and no residual activity (Knottnerus et al., 2020). This decreased the accumulation of fatty acid oxidation intermediates, which seem to have caused the changes described and is a possible explanation for the higher risk of arrhythmias in patients with this genetic disease.

Another potential molecule worth mentioning is DL-dithiothreitol (DTT), which restores thiol groups in oxidized proteins to their reduced state, somewhat inhibiting ROS effects. Treatment with DTT reversed Ca^2+^-handling disorders observed in isolated atrial cardiomyocytes after exposure to TNF-α, such as a decreased transient amplitude, increased spark frequency and duration, and increased mitochondrial ROS production (Zuo et al., 2018), suggesting that antioxidant therapy is a promising anti-arrhythmic therapy. Finally, it has also been demonstrated that bongkrekic acid, an adenine nucleotide translocase (ANT) inhibitor, has potential as an antiarrhythmic drug. In a murine model of fatty acid accumulation, fatty acids caused a partial inhibition of ANT, which was associated with increased ROS production, decreased ΔΨm, RyR2 oxidation, decreased Ca^2+^ transient amplitude, increased Ca^2+^ sparks and Ca^2+^ wave incidence, and an increased propensity to develop non-sustained ventricular tachycardia. Adding bongkrekic acid or N-acetylcysteine decreased ROS production, RyR2 oxidation, and Ca^2+^ spark frequency, as well as preserved ΔΨm and the Ca^2+^ transient amplitude and prevented the development of non-sustained ventricular tachycardia (Roussel et al., 2015).

It was previously thought that antioxidants like quercetin and resveratrol, due to their low bioavailability and non-specificity to any tissue, would make them poor candidates for new therapies (Formica and Regelson, 1995). This is backed by the fact that only limited benefits have been found when using such therapies (Askari et al., 2012; Wilson et al., 2016; Vázquez-Garza et al., 2020). Nonetheless, similar to the gene therapy described previously, the development of nanomaterials capable of packaging the components, protecting them from degradation, and directing them to a specific tissue places these often-dismissed compounds back on the table. This new way of delivering compounds has opened a wide spectrum of possible components that were disregarded in the past due to having low bioavailability or a small therapeutic window or because they were unable to pass through the cellular membrane. Furthermore, nanomaterials make it possible for two or more different compounds to be placed in the same particle, increasing the number of possibilities exponentially. A more in-depth review of nanoparticles is available (Lozano et al., 2018). Even drugs that are being used today may be packaged to provide the same or a better outcome while reducing adverse effects. It is only a matter of time before these compounds demonstrate their real potential in preserving mitochondrial integrity in a pathologic setting, such as arrhythmias. Previous clinical trials targeting ROS scavenging as an antiarrhythmic therapy have had mixed results (El-Hamamsy et al., 2007; Hicks et al., 2007; Negi et al., 2011; Rodrigo et al., 2013; Martínez-González et al., 2014; Stanger et al., 2014), some of which may be debated to be due to other effects, not only ROS scavenging (Martínez-González et al., 2014). Furthermore, a meta-analysis of clinical studies with commonly used antioxidants concluded that the studies with positive results were flawed by the small sample size and the lack of evidence of a real antioxidant effect in these patients (Violi et al., 2014). Nonetheless, there have been no clinical trials targeting mitochondrial ROS specifically, and thus the possibility of this being a new therapeutic target should not be dismissed.

#### Specific Targeting of Mitochondrial ROS Production

MitoTEMPO, a mitochondria-specific molecule, is composed of a combination of the antioxidant piperidine nitroxide tempo and the lipophilic cation triphenylphosphonium. The lipophilic cation section of the molecule enables it to pass through bilipid layers and accumulate predominantly where there is an accumulation of negative charges, which means that it is drawn toward ΔΨm. Mitochondrial concentrations of MitoTEMPO are several 100-fold when compared to cytosol. This makes it a suitable candidate to prevent excessive mitochondrial ROS accumulation and has already been tested in a murine model of diabetic cardiomyopathy. Daily dosing reduced superoxide generation in cardiomyocytes, reduced mitochondrial ROS generation, decreased apoptosis, and reduced myocardial hypertrophy, which were increased in the diabetic group without MitoTEMPO. The treatment group was also found to have a preserved ejection fraction and fractional shortening when measured by echocardiography, both of which were reduced in the diabetic group without MitoTEMPO (Ni et al., 2016). Similarly, in isolated rabbit cardiomyocytes, ISO exposure was associated with increased Ca^2+^ leakage, Ca^2+^ waves, and ROS production, as evidenced by fewer free thiols in the RyR2. These effects were partially prevented using MitoTEMPO or other ROS scavengers (Bovo et al., 2012) and were corroborated in a murine model of CPVT (Hamilton et al., 2020). MitoTEMPO has also been described as being able to prevent sudden cardiac death and ventricular arrhythmias in an HF guinea pig model, as well as prevent remodeling (Dey et al., 2018). Other mitochondria-specific molecules have also produced similar results. In an I/R model, peptides SS-31 and SS-20 reduced infarct size and severity, as well as the duration of reperfusion arrhythmias and lipid peroxidation, most likely by scavenging and reducing ROS production (Cho et al., 2007). Observations of human ventricular myocytes from patients with end-stage HF further support this idea. In this condition, it has been shown that RyR2s become uncoupled from nearby T-tubules and prone to spontaneous activation. Treatment with mitochondrial ROS scavengers reduces the receptor’s hyperactivity and spontaneous Ca^2+^ wave formation under adrenergic stimulation (Dries et al., 2018). These compounds are, therefore, another viable treatment option to reduce mitochondrial ROS accumulation and subsequent mitochondrial dysfunction.

### Modulation of mPTP Opening

Despite its elusive composition, the mPTP is strongly regulated by cyclophilin D (CypD), the VDAC, ANT, the phosphate carrier, ATP synthase, and the translocator protein (TSPO), formerly known as the peripheral benzodiazepine receptor (Alves-Figueiredo et al., 2021). A causative relationship between ROS-induced mPTP opening and ΔΨ*_m_* depolarization has also been demonstrated in numerous studies. In particular, there is some evidence that the mPTP may also be regulated by TSPO through interaction with the other components (Zulian et al., 2011). Using TSPO ligands has been shown to inhibit ΔΨ*_m_* depolarization after excessive ROS exposure (Leducq et al., 2003). TSPO became known as a potential therapeutic target when it was shown to activate a channel in the IMM, the inner membrane anion channel (IMAC). This channel is described as being able to depolarize ΔΨ*_m_* in an mPTP-independent way, causing oscillations in ΔΨ*_m_* and intermittent sarcKATP channel activation (Aon et al., 2003). These oscillations cause variation in the APD, which promotes a reentry pathway to the electrical stimuli. Inhibiting TSPO, therefore, has the potential to cover two different pathways to mitochondrial dysfunction, IMAC-mediated and mPTP-mediated (Gambardella et al., 2017), making this protein a potential therapeutic target. Although studies have shown that TSPO ligands can reduce ROS levels and abolish metabolic and electrophysiological changes, they have yet to be tested in a clinical setting (Akar et al., 2005). Protection against mitochondrial depolarization has been observed to translate into reduced APD shortening and inexcitability in a dose-dependent manner. However, the use of FGIN-1-27, an IMAC activator, promoted faster shortening of the APD and resulted in an earlier loss of conduction during reperfusion (Akar et al., 2005). Hearts treated with an IMAC activator prior to ischemia are more responsive to it. High-resolution optical mapping demonstrated small areas of conduction blockage during early ischemia that persisted throughout the whole recording, making it more suitable for reentry, which caused VF. IMAC inhibition stabilized ΔΨm *in vitro*, suppressed reperfusion arrhythmias, and promoted faster AP recovery (Akar et al., 2005). The protective effect on AP recovery was also demonstrated in a rabbit model of I/R (Brown et al., 2008). Notably, the antiarrhythmic effect afforded by TSPO ligands was not evident in hearts treated with the mPTP desensitizer CsA. This finding indicates that IMAC and mPTP opening are primary mitochondrial mediators of acute postischemic arrhythmias. Finally, mPTP opening, which can be viewed as an irreversible loss of mitochondrial function, leads to cell death. Over time, cardiomyocytes are replaced with fibrotic tissue, which generates a physical barrier that prevents proper stimulation of the myocardium in an orderly manner. This pathological remodeling creates possible paths for re-entry arrhythmias.

As mentioned previously, mPTP opening is the process by which mitochondrial membrane integrity is permanently lost, and content from the mitochondrial matrix flows toward the cytosol. Thus, the mitochondria are no longer able to maintain their ΔΨm, ATP is unable to be synthesized, and there is a subsequent loss of ionic balance. Between the leaked components that are translocated to the cytosol, cytochrome C, which normally takes part in the ETC, starts a signaling cascade for cell apoptosis. Cardiomyocyte death may be the most challenging arrhythmogenic process to deal with, as cardiomyocytes do not regenerate and are replaced with fibrotic tissue. This fibrotic tissue not only reduces the myocardium’s pool of available contractile units to create effective blood pumping but also creates zones in which electrical signals do not travel at the proper speed, paving the way for disorganized contraction. CsA, a well-known immunosuppressor, was found to be a potent mPTP inhibitor many years ago (Crompton et al., 1988). Later, it was found that the compound’s inhibitory effect on the mPTP is achieved by inhibiting CypD, a component required for mPTP opening (Davidson and Halestrap, 1990; Griffiths and Halestrap, 1991). It was discovered that inhibiting mPTP with CsA reduces I/R-induced arrhythmias by preserving cardiac tissue from suffering edema and reducing cardiomyocyte death, presumably by protecting mitochondria from Ca^2+^-induced damage (Arteaga et al., 1992). mPTP inhibition was then corroborated as a potential strategy when another inhibitor, octylguanidine, was observed to protect against arrhythmias and edema caused by I/R, as well as maintain blood pressure and mitochondrial function (Parra et al., 2005). The effect was later confirmed in a hyperthyroid murine model undergoing I/R. Octylguanidine protects against arrhythmias by maintaining mitochondrial functioning and cardiac hemodynamic parameters and reducing inflammatory cytokines (Pavón et al., 2009). Another mPTP inhibitor, citicoline, was also useful in preventing arrhythmia and blood pressure drop during I/R. Mitochondria maintained adequate ΔΨm, high respiratory control, cis-aconitase enzyme activity, and mDNA integrity (Hernández-Esquivel et al., 2014). It was then shown that mPTP inhibition by CsA administration was related to reduced infarct size in a murine model of I/R (Xie and Yu, 2007). CsA was also shown to reduce hypertrophy and arrhythmia susceptibility in a canine model of chronic atrioventricular blockage. CsA administration was not associated with reducing the electrical remodeling observed in the model, as indicated by a prolonged effective refractory period similar to the no-treatment group, although it did reduce the thickness of free and septal ventricular walls. Nonetheless, the CsA group showed no susceptibility to drug-induced polymorphic ventricular tachycardia (Schreiner et al., 2004). This led to the conclusion that electrical remodeling is only partially responsible for the susceptibility to arrhythmias seen in the model and that hypertrophy is also a necessary substrate for arrhythmogenesis. However, more recent studies found no protective effect when using CsA and measuring mPTP opening *in vivo* in a VF murine model. There were no differences in troponin I, cytochrome c, or NAD^+^ levels when compared to the group treated with just the vehicle, as well as in hemodynamic and left ventricular (LV) function parameters (Ayoub et al., 2017). Although it could be argued that CsA was administered at half the dosing concentration used in previous studies and was administered for only 5 min before inducing VF or before starting the resuscitation protocol. Similar results were found when comparing 4′-chlorodiazepam (4-ClDzp), a ligand for TSPO, to CsA in a rabbit I/R model. CsA offered no protection against arrhythmias, in contrast to 4-ClDzp, which preserved normal cardiac functioning when given as an infusion throughout the I/R or as a single bolus before reperfusion (Brown et al., 2008). Another study utilized guinea pig hearts in an I/R protocol. 4-ClDzp preserved ΔΨm, reduced AP shortening, and prevented reperfusion arrhythmias. Recovery was remarkably higher than in the group treated with CsA (Akar et al., 2005). This evidence raises the possibility of yet another potential target for new anti-arrhythmic therapies, and some authors have even proposed that the mediator of post-ischemic arrhythmias is the IMAC, which can be regulated via TSPO, rather than the mPTP (Motloch et al., 2015). However, a CypD KO murine model proved that the mPTP does, indeed, play a role in arrhythmogenesis. At the cellular level, cardiomyocytes from CypD KO mice were much less likely to undergo mPTP opening upon the addition of FCCP. Likewise, the incidence of Ca^2+^ waves and Ca^2+^ alternans was much lower than in their WT counterparts. Similar results were also obtained when CsA was added to WT cardiomyocytes. At the *ex vivo* heart level, ECGs showed ST-T wave alternans in the WT group, but the alternans were absent in the CypD KO group and only observed in one heart in the CsA group. Arrhythmia scores were consistently much lower upon FCCP addition or I/R (Gordan et al., 2016). Using the same CypD KO model, arrhythmia susceptibility was tested under an iron overload protocol. Again, WT cardiomyocytes presented with increased ROS production, diminished ΔΨm, and frequent Ca^2+^ waves, while *ex vivo* hearts presented with arrhythmias upon stimulation. Although the CypD KO group presented with similar ROS production and decreased ΔΨm, its Ca^2+^ waves were significantly less frequent, and arrhythmia scores were lower. Similar results were found when the WT group was treated with CsA (Gordan et al., 2020). Although there may be contradictory evidence, CsA is still thought to be a molecule with therapeutic potential.

These findings have led to the use of CsA as a positive control for mitochondrial protection against mPTP, with which other treatments can be compared. However, when CsA was used as a treatment for out-of-hospital cardiac arrest in the CYRUS trial, there was no difference in organ failure, survival at hospital admission, survival at 24 h, survival at hospital discharge, or favorable neurologic outcomes when compared to the control group (Argaud et al., 2016). This finding led to the reasoning that in clinical practice, patients who are susceptible to cardiac arrest or have a baseline cardiopathy have already undergone adaptations in their cardiac tissue that make them less susceptible to mPTP protection with CsA, unlike murine models of ischemia-reperfusion that use healthy and young animals in both groups. After integrating the information presented here, it is possible that mPTP inhibition delays irreversible mitochondrial damage, giving the cardiomyocyte time to fix the underlying cause. It may be helpful in a young, healthy animal model in which the insult is temporal and where the cardiac tissue can deal with the ROS generated after reperfusion, given the extra time CsA provides before mPTP opening. However, in the context of a patient with severe atherosclerosis or HF, which is more commonly the case in patients with a high risk of arrhythmias, there is already increased ROS production and Ca^2+^ overload, which decreased the cell’s resources considerably before ischemia. Delaying mPTP opening would have little effect in preserving cardiac tissue since the cardiomyocyte is unable to re-establish homeostatic conditions, even if given more time with CsA. Nonetheless, there is still the possibility that CsA, used in combination with another therapy, could synergize by delaying mPTP opening, and other therapies could take effect in cardiomyocytes, which would not have had enough time for recovery otherwise.

### Regulation of Mitochondrial Quality Control

Excessive mitochondrial fission has been observed to promote cell death in I/R models. Mdivi-1, a fission inhibitor that has been shown to prevent apoptosis, and M1, a cell-permeable phenylhydrazone fusion promoter, were administered in an I/R murine model (Maneechote et al., 2018, 2019). Both treatments significantly reduced arrhythmias after reperfusion when given prior to ischemia, as well as reduced the decline in hemodynamic parameters. Preventing excessive fission with the subsequent reduction in ΔΨm and ATP synthesis and increasing ROS generation is yet another potential therapeutic strategy to prevent arrhythmia generation in high-risk patients. As stated previously, a pressure-overload HF murine model was shown to have a blockade in the cell’s mitophagic function, leading to damaged mitochondria accumulation. When given the mCU inhibitor RuR or Ru_360_, the mitophagic profile improved to reveal no blockade, along with improvement in cardiac function, reduction in ventricular asynchrony, and preserved mitochondrial integrity (Yu et al., 2018). This finding indicates that some therapies, such as mCU modulation, have effects at different levels and signaling pathways. Further research is needed to elucidate the mechanisms by which this happens to better understand the effects that such strategies have at the mitochondrial and cellular levels.

### Maintaining Energetic Balance With High-Energy Phosphate Analogs

A central point of Ca^2+^ dysregulation is the energetic deficiency that ensues given the lower ATP production by dysfunctional mitochondria, accompanied by higher energetic demand from a less efficient Ca^2+^ removal system. Thus, replenishing the energetic reserve with exogenous molecules is another possible approach. In this regard, cyclocreatine, a creatine analog that is permeable to membranes, has been used mainly as a therapy for I/R injury. Prior treatment with cyclocreatine in the diet has been shown to preserve ATP levels longer during ischemia in chicken hearts (Turner and Walker, 1985) when measured by conventional techniques, as well as in rat hearts when measured via nuclear magnetic resonance imaging (Osbakken et al., 1992). Cyclocreatine has also been demonstrated to have anti-inflammatory effects in isolated heart (Elgebaly et al., 1990) and *in vivo* (Elgebaly et al., 1993) models of ischemia. Recovery of cardiac function after cold storage has also been improved when hearts were previously treated with cyclocreatine phosphate (Elgebaly et al., 1994), as well as in models of cardiopulmonary bypass (Houser et al., 1995). Previous studies have only been conducted in the context of ischemia and in clinical trials using other high-energy phosphate analogs without favorable results in HF or myocardial infarction patients (Horjus et al., 2011). However, it may still be worthwhile to research the effects of compounds like cyclocreatine in arrhythmia models, as it has been stated that the failure of these substances to preserve ATP levels and, thus, cardiac function may be due to kinetic differences in their ability to become a substrate for creatine kinase and their phosphoryl group transfer potential. Although monotherapy with high-energy phosphate analogs may not be effective in preventing arrhythmias, it can still potentiate the effects of other therapies by increasing and preserving the heart energy pool.

### MitoK_ATP_ Channel Modulators

MitoK_ATP_ channels are structures within the IMM that, upon opening, partially dissipate ΔΨm. This partial depolarization diminishes the driving force for Ca^2+^ entry and thus prevents Ca^2+^ overload and generates ROS, which have been associated with the protection conferred by ischemic preconditioning (Pain et al., 2000). Agonists of this channel have been assessed as a possible therapeutic approach. 4-carboxy phenyl-isothiocyanate (4CPI), a hydrogen sulfide (H2S) pro-drug and mitoK_ATP_ agonist, reduced tissue injury and ROS production, prevented arrhythmias, and improved recovery upon reperfusion in murine models of I/R; these effects were lost when 5-hydroxydecanoic acid, a mitoK_ATP_ blocker, was added (Testai et al., 2016). Compound A, another mitoK_ATP_ agonist, has also been proven to reduce reperfusion arrhythmias in I/R, with effects even more pronounced than diazoxide, a more commonly used mitoK_ATP_ agonist. Unfortunately, these effects were not more effective when used in combination with a sarcolemmal K_ATP_ blocker (Gonca et al., 2016). Even sevoflurane, a commonly used anesthetic, has been demonstrated to have protective effects dependent on mitoK_ATP_ opening. Preconditioning isolated rat hearts with sevoflurane was demonstrated to have protective effects upon I/R, as evidenced by smaller infarct size, reduced cardiac troponin I levels, upregulated PKC, and downregulated caspase 8. This protection was lost when hearts were pretreated with 5-hydroxydecanoic acid, suggesting the role of mitoK_ATP_ in the protective effects of sevoflurane (Wang et al., 2015). While these results seem promising, more studies are needed, mainly in other models susceptible to generating arrhythmias, to obtain more solid evidence that mitoK_ATP_ is a potential antiarrhythmic therapy.

In the last few years, there has been considerable advancement in understanding the molecular mechanisms of cardiac disease. This has laid a path to the development of better therapeutic strategies that were first ignored or disregarded as potentially ineffective. It has also led to the discontinuation of practices that were once thought to be helpful but then proved to be either neutral or damaging. It may be time to expand our viewpoint and explore the crosstalk that takes place between organelles and seems to orchestrate Ca^2+^ homeostasis rather than focus on a single cellular component for a new therapy to prevent sudden cardiac death. This may be the right way to prevent arrhythmogenesis, by tackling the central mechanism that leads to different cellular malfunctions.

### Mitochondrial-SR Interaction

#### CAMKII Modulation to Prevent SR Ca^2+^ Leaks Into Mitochondria

Ca^2+^/calmodulin-dependent protein kinase II (CAMKII) is an enzyme normally activated by Ca^2+^-calmodulin and phosphorylates a wide range of proteins, including RyR2, PLB, and LTCCs. Increased CAMKII activity has been observed in pathological processes, such as cardiac hypertrophy and HF (Kirchhefer et al., 1999; Zhang et al., 2003). Whether a higher phosphorylated state of the RyR2 leads to a subsequently higher opening probability in diastolic Ca^2+^ concentrations, the generation of Ca^2+^ sparks, lower SR Ca^2+^ content, and a negative force-frequency relationship (Ai et al., 2005; MacDonnell et al., 2008; Kushnir et al., 2010) remains controversial. Unsurprisingly, CAMKII has been a potential target in the prevention of hypertrophy, arrhythmias, and HF. Modulating its activity has led to interesting results regarding cardiac tissue protection. KN-93, the most well-known CAMKII inhibitor, has been shown to prevent AF in mice with a gain-of-function mutation in the RyR2 after fast atrial pacing (Chelu et al., 2009). AIP, another CAMKII inhibitor, has also been shown to prevent afterdepolarization events in isolated cardiomyocytes in a murine model overexpressing CaMKIIδ_C_. Additionally, KN-93 administration *in vivo* prevented ISO-induced arrhythmias (Sag et al., 2009). In a murine model of diabetic hyperglycemia, KN-93 administration reduced premature ventricular contractions, as well as Ca^2+^ sparks, and was further supported with similar findings from diabetic failing heart donors (Erickson et al., 2013). This treatment strategy has also proved to be effective in a murine model expressing constitutively active Ca^2+^-dependent phosphatase calcineurin (CAN), in which KN-93 administration reduced arrhythmia susceptibility, as well as improved ventricular cardiomyocyte contractile functioning (Khoo et al., 2006). Similarly, an impaired glucose tolerance murine model was found to have arrhythmias and arrhythmogenic events related to hyperphosphorylation of Ca^2+^ handling proteins in isolated ventricular cardiomyocytes, which were prevented when a transgenic model that expressed AIP was submitted to the same impaired glucose tolerance protocol (Federico et al., 2019). Furthermore, CaMKII also regulates mitochondrial functioning by controlling fatty acid oxidation through palmitoyl-CoA transferase I (Sharma et al., 2010) and Ca^2+^-dependent dehydrogenases via the mCU (Joiner et al., 2012); however, some studies do not support this conclusion (Nickel et al., 2020). CaMKII might enhance mCU activity by phosphorylating the mCU’s N-terminals and concomitantly lead to mPTP opening by mitochondrial Ca^2+^ overload and, ultimately, cell death upon I/R injury or dilated cardiomyopathy (Joiner et al., 2012). Chronic CaMKII activation has also been shown to be involved in mitochondrial gene reprogramming, leading to mitochondrial dysfunction and increased oxidative stress in cardiac hypertrophy (Joiner and Koval, 2014). CaMKII also mediates Drp1 phosphorylation, which has been shown to increase the frequency of mPTP opening upon chronic β-adrenergic stimulation (Xu et al., 2016). Although the use of a CaMKII inhibitor has not been demonstrated in mitochondrial physiology, KN-93 administration has also been used in a murine model of CPVT. Arrhythmias were abolished when treated with KN-93, and the spark frequency was reduced in isolated cardiomyocytes when treated with ISO (Liu et al., 2011). These findings are further supported by cardiomyocytes derived from induced pluripotent stem cells of CPVT patients presenting with similar findings. All this evidence suggests that CAMKII activation plays a major role not only in arrhythmogenesis but also in other cardiac pathologies, such as HF progression. Further studies regarding the development of a more specific component with a much higher affinity could bring us closer to the development of a new therapeutic regime.

#### RyR2 Stabilizers to Reduce Mitochondrial Ca^2+^ Overload

Dantrolene is a muscle relaxant known widely for its use in treating malignant hyperthermia. This condition arises when a patient with a mutation in the RyR2 is exposed to inhaled halogenated anesthetics. This mutation makes the RyR2 susceptible to massive Ca^2+^ release, which is associated with muscle breakdown, elevated serum creatinine kinase, hypotension, hyperthermia, tachycardia, and intraoperative death. Its mechanism of action was unknown until a few years ago, when it was found to bind the RyR2 and stabilize domains within the receptor (Paul-Pletzer et al., 2002; Kobayashi et al., 2005). Its mechanism of action seems suitable for preventing stochastic Ca^2+^ release during diastole and has been studied in various scenarios. It has shown therapeutic potential in a murine model of CPVT, in which mice were subjected to a knock-in procedure of a known mutation that causes the pathology. Transgenic mice presented with ventricular tachycardia when stimulated with epinephrine or by running on a treadmill. Pretreatment with dantrolene for 7–10 days inhibited VT. In isolated cardiomyocytes, the spark frequency was also diminished (Kobayashi et al., 2010). In an *ex vivo* model of long-QT syndrome, rabbit hearts were infused with erythromycin or veratridine to mimic the condition. When the potassium concentration was lowered, the hearts presented with early EADs and polymorphic ventricular tachycardia. Infusion with dantrolene resulted in a significant reduction of EADs and polymorphic VT (Frommeyer et al., 2018). Dantrolene also regulates mitochondrial-SR interconnection in cardiomyocytes from a model of HF with increased stochastic RyR2 activity. The author identified that spontaneous Ca^2+^ waves, which can subsequently propagate and trigger organ-wide arrhythmia, were increased in the presence of the mCU agonist (Hamilton et al., 2018). Under these conditions, kaempferol-treated ventricular myocytes showed a significant increase in the percentage of cells presenting with spontaneous Ca^2+^ waves. Interestingly, stabilization of the RyR2 with dantrolene reduced the proarrhythmic effects of kaempferol on Ca^2+^ handling (Hamilton et al., 2018). In this context, some authors (Beutner et al., 2001) have identified a RyR2-like protein in the IMM. This elusive Ca^2+^ transporter demonstrates that it might be possible to modulate dantrolene-sensitive mitochondrial functions, such as Ca^2+^-dependent O_2_ consumption, Ca^2+^-dependent mPTP and swelling, and cytochrome c release (Beutner et al., 2001), with a direct effect on mitochondrial RyR2.

Dantrolene has also been used to treat AF in a sheep model of atrial infarction. Left atrial myocardial infarction was induced by ligating the atrial branch of the left anterior descending coronary artery. After the procedure, numerous episodes of AF appeared. It was noted that these episodes were produced by spontaneous focal discharges occurring in the zone between the infarcted and normal tissue and were inducible with ISO. Dantrolene administration after the procedure prevented the focal discharges and AF, presumably by maintaining a proper RyR2 response to calmodulin (Avula et al., 2018). It has also been compared to amiodarone in VF treatment using a pig model of cardiac arrest. Under anesthesia, VF was induced by an electrode in the right ventricle and left untreated for 8 min. Afterward, chest compressions and ventilation were started, along with the administration of either amiodarone, dantrolene, or saline. After 4 min of resuscitation, defibrillation was attempted. The rates of return to spontaneous circulation and hemodynamic stability were not statistically different in the dantrolene and amiodarone groups (Wiesmann et al., 2017). This finding implies that dantrolene is as effective as other anti-arrhythmic drugs currently in use. In a more clinical setting, dantrolene has shown efficacy in reducing spark frequency, diastolic SR Ca^2+^ leakage, Ca^2+^ waves, and spontaneous Ca^2+^ transients in isolated human cardiomyocytes from patients with AF or end-stage HF (Hartmann et al., 2017). Similarly, a small group of patients with CPVT underwent an exercise stress test, and ECG was recorded before and after dantrolene administration. Premature ventricular complexes were reduced in patients enrolled in the study. Similarly, cardiomyocytes derived from induced pluripotent stem cells from fibroblasts from these patients were stimulated with epinephrine, and Ca^2+^ transients were recorded. Dantrolene, again, abolished most of the abnormalities registered in patients (Penttinen et al., 2015). Notably, some patients who responded to treatment had mutations in the N-terminal or central cytosolic region of the RyR2 protein, while the non-responders had their mutations in or near the transmembrane domain. This brings attention to the fact that, at least in the case of CPVT, the baseline pathology needs to be checked to ensure pharmaceutical effectiveness.

However, dissociation of this protein with the RyR2 macromolecular complex has been described in a variety of cardiac pathologies, such as HF, hereditary forms of exercise-induced arrhythmias, and sudden cardiac death. Dissociation of this protein causes the RyR2 to become more sensitized to Ca^2+^-dependent activation, increasing diastolic Ca^2+^ leakage, impairing Ca^2+^ cycling, and decreasing contractility (Lehnart et al., 2004b). Increasing the affinity of calstabin2 to the RyR2 macromolecular complex with the 1,4-benzothiazepine derivative molecule JTV519 or S107 has been useful in preventing arrhythmias and sudden cardiac death. In one of the first experiments, JTV519 was found to correct abnormal RyR2 channel gating in dogs with induced HF (Kohno et al., 2003). In another study, calstabin2^±^ mice presented with ventricular tachycardia and sudden cardiac death when put under strenuous exercise. However, these events were prevented entirely when treated with JTV519 (Wehrens et al., 2004). Treatment with JTV519 was associated with a decreased opening probability of individual RyR2 channels when subjected to diastolic Ca^2+^ concentrations compared to the untreated group, which had abnormally high opening probabilities. Calstabin2^–/–^ mice, however, exhibited no protection from JTV519, demonstrating that calstabin2 must be present for JTV519 to prevent stochastic RyR2 channel opening. This molecule has also been shown to stabilize RyR2 opening in the context of mutations found in CPVT patients. Treatment with JTV519 was found to increase the association between calstabin2 and the RyR2 and normalize the channel’s functioning (Lehnart et al., 2004a). This was recently demonstrated using 3D-engineered heart tissue transfected to express channelrhodopsin-2, an ion channel activated by light. Under a chronic optical tachypacing protocol, the tissue remodeling showed a propensity for tachycardic episodes when submitted to a burst pacing protocol. This propensity was abolished when JTV519 was administered (Lemme et al., 2019). In addition, in guinea-pig ventricular muscles subjected to no-flow ischemia followed by reperfusion, JTV519 administration significantly improved post-ischemic contractile recovery. However, the potential benefit of JTV519 administration in models of I/R injury was blocked with 5-HD, thereby suggesting that the activation of mitochondrial K_ATP_ channels mediates the cardioprotective effects of JTV519 (Ito et al., 2000).

Given the non-specific effects that JTV519 could have on other ion channels, a new molecule, S107, was initially developed as a treatment to increase skeletal muscle exercise capacity (Bellinger et al., 2008). However, this new molecule prevented arrhythmias and seizures in mice harboring a CPVT mutation in the RyR2 (Lehnart et al., 2008). The protective effect was also observed in a Duchenne muscular dystrophy (DMD) murine model. DMD mice were found to have structural remodeling in their cardiac tissue. This remodeling predisposed the model to ventricular arrhythmias, which were prevented completely with S107 administration (Fauconnier et al., 2010). S107 was also able to suppress AF in different murine models with *knock-in* mutations known to cause CPVT in humans (Shan et al., 2012). In another study using cardiomyocytes derived from induced pluripotent stem cells from a human patient with CPVT, pre-incubation with S107 prior to ISO exposure dramatically reduced the percentage of cells that developed DADs (Sasaki et al., 2016). Finally, a new optogenetic animal model using nematodes with mutations in proteins analogous to mutations in the RyR2 or calsequestrin in CPVT patients displayed similar muscular dysfunction findings upon a stress challenge with a faster pacing rate; muscular dysfunction was prevented with the use of S107 (Fischer et al., 2017).

#### SERCA Modulation to Improve ECEC

Numerous studies have shown that SERCA’s functionality is reduced in common cardiac pathologies and conditions prone to developing arrhythmias. Such is the case of AF, in which reduced SERCA expression has been described in the peripheral blood cells of patients with AF, and these levels of expression can be used to predict the clinical response to treatment, such as epicardial thoracoscopic PV isolation (Sardu et al., 2020). Unsurprisingly, increasing SERCA’s capacity to pump Ca^2+^ into the SR to increase SR Ca^2+^ content, which, in turn, increases the amount of Ca^2+^ released during CICR and translates into generating a greater contractile force, along with a lower probability of stochastic RyR2 opening, by decreasing cytosolic Ca^2+^ concentrations during diastole. This is one of the most studied approaches to treating cardiac dysfunction and has been assessed from different viewpoints. A small group of patients with advanced HF received an intracoronary infusion of an adeno-associated viral vector (AAV) containing genetic material that coded for SER-CA2a. These patients presented with improvements in symptomatic, functional, biomarker, and LV functional parameters, along with no significant adverse effects in other organs (Jaski et al., 2009). Notably, two patients who did not experience clinical improvement despite the therapy already had circulating antibodies against the viral vector before transfusion. In a phase 2a trial, 39 patients received an intracoronary infusion of the AAV. The same parameters were assessed. Again, the treatment group presented with improvement, an increased time from therapy to the occurrence of clinical events related to HF, and a decreased frequency of cardiovascular events in the following 12 months (Jessup et al., 2011). However, when the phase 2b trial was conducted, unfortunately, after a median follow-up of 17.5 months, the treatment group showed no improvement in time to recurrent events or mortality when compared to placebo (Greenberg et al., 2016). The reason these results were so different from previous studies remains unclear. It is hypothesized that the previous studies’ results were affected by chance, considering the small number of patients, or the formulation used in the CUPID 2 trial was not effective enough. Although the outcome was unexpected, it at least proved gene therapy is a viable option, as there were no major adverse effects when compared to placebo. Furthermore, the trial provides insight into what needs further development before a second attempt to implement gene therapy is performed.

Another strategy used to enhance SERCA’s Ca^2+^ pumping capacity is to functionally reduce its inhibitor protein, PLB. This strategy has been studied with a PLB KO murine model. This model presented with a similar magnitude in the LTCC current when compared to wild-type, although with a faster decay. Regarding differences, a larger AP that decayed faster had greater SR Ca^2+^ content, better ECC (measured as Δ cytosolic Ca^2+^ concentration/LTCC current), and more frequent and greater Ca^2+^ sparks were noted, although the decay times were similar (Santana et al., 1997). This model also presented with better diastolic functioning in Doppler and color M-mode echocardiography (Schmidt et al., 2002). Furthermore, combining a SERCA overexpression model with a PLB KO model resulted in an even more enhanced cardiac state. Mice with both genetic modifications presented with higher maximal rates of contraction and relaxation and lower Ca^2+^ transient decay times when compared to groups with either single mutation. No histological or pathological changes were found in the double transgenic model (Zhao et al., 2003). This demonstrates the synergy achieved when enhancing SERCA’s functionality from two different approaches, making it possible to simultaneously address SERCA’s dysfunction with different treatments. Interestingly, these transgenic mice exhibited increased oxygen consumption to meet the demand for increased ATP consumption. Higher levels of mitochondrial oxygen consumption were associated with increased Ca^2+^-dependent pyruvate dehydrogenase activity. These findings suggest that the ablation of PLB requires metabolic adaptations to establish proper ECEC (Chu et al., 1996).

Concerns about enhancing SERCA in a dysfunctional setting have been expressed. Because the RyR2 is commonly dysfunctional in most settings where SERCA is less efficient in removing diastolic Ca^2+^, it is implied that increasing SR Ca^2+^ without addressing the increased opening probability of the RyR2 could result in an enhanced spark frequency and higher Ca^2+^ wave propagation, caused by RyR2 opening due to increased luminal Ca^2+^ sensibility, with subsequent arrhythmia development. Nonetheless, studies involving the enhancement of SERCA’s functioning in such settings, including HF models and patients with advanced HF, have not shown such adverse effects. This may be due to SERCA’s enhanced efficiency being more beneficial by reducing diastolic Ca^2+^, as it seems that the RyR2 still needs cytosolic Ca^2+^ for stochastic opening, even under conditions of increased sensitivity to luminal Ca^2+^ content, since reaching a certain SR Ca^2+^ threshold seems insufficient for diastolic RyR2 opening (Belevych et al., 2012).

## Final Remarks

The SR and mitochondria engage in constant, intimate communication to properly respond to workload and metabolic needs. However, their interconnectedness leaves both organelles vulnerable to malfunction if one should have its safety mechanisms overrun. In this case, conditions that favor Ca^2+^ overload, such as chronic adrenergic stimulation, could elevate the diastolic mitochondrial Ca^2+^ concentration to the point at which Ca^2+^ overload ensues. This overload increases ROS production and, once the antioxidant system is overrun, causes ΔΨm loss (by mPTP and IMAC-mediated mechanisms), decreased ATP production, and mPTP opening. Furthermore, increased ROS oxidates the mCU, along with possible phosphorylation from AMPK, secondary to low ATP synthesis, which further increases Ca^2+^ transport into the matrix, creating a positive feedback loop for more ROS production. ROS can also affect nearby structures, such as SERCA, which decreases its effectiveness in pumping Ca^2+^ back into the SR. This translates into reduced SR content and higher cytoplasmic Ca^2+^ concentrations, reducing contractility. Ca^2+^ can then activate CAMKII, which can also be activated by mitochondrial ROS. CAMKII can then autophosphorylate itself to stay in a permanent active form. RyR2 is activated by CAMKII phosphorylation and ROS, which causes its regulator protein, calstabin, to detach from the RyR2 complex. This increased sensibilization promotes stochastic opening with subsequent Ca^2+^ leaks and Ca^2+^ waves, further increasing mitochondrial Ca^2+^ transport. On the sarcolemma, the oxidation of LTCCs increase the amount of Ca^2+^ that enters the cardiomyocyte upon activation. A reduction in the ATP concentration caused by mitochondrial dysfunction opens sarcKATP channels, reducing the cardiomyocyte’s bathmotropism and dromotropism. The constantly high Ca^2+^ concentration also activates NCX, slowly depolarizing the cell and promoting unsolicited depolarizations and APs. Finally, connexin 43 translocation or Panx1 opening due to increased Ca^2+^ and ROS translocates ATP and adenine into the extracellular matrix, where they activate signaling pathways for TGF-β1 expression, with subsequent fibrosis. This represents two pathways to cardiac tissue fibrosis: cell death-dependent and TGF-β1-dependent. Both have the same outcomes, creating patches of slow conduction and promoting reentry. All these effects make a suitable environment for conduction dysfunction and arrhythmia generation. Up until now, classic antiarrhythmic drugs’ main mechanism known to prevent arrhythmias does not include the mitochondrial-SR interconnection. However, they have effects on either of these organelles (Sugiyama et al., 1985; Sano et al., 1990; Deng and Zhang, 1993; Tsutsumi et al., 2001; Afanas’ev et al., 2002; Ugdyzhekova et al., 2005; Wang et al., 2007; Bannister et al., 2015; Kryshtal et al., 2020). Flecainide can inhibit RyR2 opening, although its relevance in preventing arrhythmias is still controversial (Bannister et al., 2015; Kryshtal et al., 2020). Lidocaine might inhibit mitoK_ATP_ channels, as shown in isolated cardiomyocytes utilizing a mitochondrial redox state reporter as a surrogate for mitoK_ATP_ opening (Tsutsumi et al., 2001), and prevents mitochondrial Ca^2+^ overload in a model of closed-chest VF and resuscitation (Wang et al., 2007), but the widely reported mechanism of action of Lidocaine is prolonging the inactivation of the fast voltage-gated Na^+^ channels, inhibiting spontaneous depolarization (Sheu and Lederer, 1985). Amiodarone was found to preserve mitochondrial respiration after ischemia in a model of ischemia-induced ventricular arrhythmias (Sano et al., 1990) and was shown to potentiate the SR’s ability to accumulate Ca^2+^ in either rat or isolated myocardial strips from coronary heart disease patients (Afanas’ev et al., 2002; Ugdyzhekova et al., 2005). However, its reported action is by inhibiting the I_Kr_ current, prolonging phase 3 of the AP. Finally, Verapamil blocks voltage-dependent Ca^2+^ channels, decreasing impulse conduction through the AV node, but has been reported that indirectly prevents mitochondrial Ca^2+^ accumulation (Sugiyama et al., 1985) and reduce mitochondrial oxidative stress in the context of ischemia-reperfusion by a lower MDA content in mitochondria (Deng and Zhang, 1993). These effects could be additional antiarrhythmic mechanisms that should be further addressed to fully understand and assess their physiological relevance for future antiarrhythmic therapy developing.

## Conclusion

In the last few years, there have been considerable advancements in understanding the molecular mechanisms of cardiac disease, especially arrhythmogenesis. These advancements laid the path for developing better therapeutic strategies that were first ignored or disregarded as potentially ineffective. Gaining a better understanding of the molecular mechanisms involved in arrhythmia generation provides insights that could lead to new therapeutic strategies. It may be time to expand our viewpoint and explore the crosstalk taking place between mitochondria-SR interconnection that seem to orchestrate Ca^2+^ homeostasis instead of focusing on a single cellular component. This may be the most effective way to prevent arrhythmogenesis, by tackling the central mechanism that leads to different cellular malfunctions.

## Author Contributions

GG-R conception and designed the review. FS-R, RR-M, and GG-R analyzed the literature and drafted the manuscript. GG-R and RR-M contributed to critial review of the manuscript. This work was submitted in partial fulfillment of the requirements for the Ph.D. degree (FS-R) for the Doctorate in Biomedical Sciences. Tecnologico de Monterrey. All authors contributed to the article and approved the submitted version.

## Conflict of Interest

The authors declare that the research was conducted in the absence of any commercial or financial relationships that could be construed as a potential conflict of interest.

## References

[B1] Adabag A. S. Luepker R. V. Roger V. L. Gersh B. J. (2010). Sudden cardiac death: epidemiology and risk factors. *Nat. Rev. Cardiol.* 7 216–225. 20142817 10.1038/nrcardio.2010.3 PMC5014372

[B2] Afanas’ev S. A. Lukavskaya I. A. Kandinskii M. L. Medvedev M. A. (2002). Effect of amiodarone on functional state of sarcoplasmic reticulum in rat myocardium. *Bull. Exp. Biol. Med.* 133 205–207. 12360330 10.1023/a:1015832710818

[B3] Ai X. Curran J. W. Shannon T. R. Bers D. M. Pogwizd S. M. (2005). Ca2+/calmodulin-dependent protein kinase modulates cardiac ryanodine receptor phosphorylation and sarcoplasmic reticulum Ca2+ leak in heart failure. *Circ. Res.* 97 1314–1322. 10.1161/01.res.0000194329.41863.89 16269653

[B4] Akar F. G. Aon M. A. Tomaselli G. F. O’Rourke B. (2005). The mitochondrial origin of postischemic arrhythmias. *J. Clin. Invest.* 115 3527–3535. 10.1172/jci25371 16284648 PMC1280968

[B5] Alfadda A. A. Sallam R. M. (2012). Reactive oxygen species in health and disease. *J. Biomed. Biotechnol.* 2012:936486. 10.1155/2012/936486 PMC3424049 22927725

[B6] Alves-Figueiredo H. Silva-Platas C. Lozano O. Vázquez-Garza E. Guerrero-Beltrán C. E. Zarain-Herzberg A. (2021). A systematic review of post-translational modifications in the mitochondrial permeability transition pore complex associated with cardiac diseases. *Biochim. Biophys. Acta Mol. Basis Dis.* 1867:165992. 10.1016/j.bbadis.2020.165992 33091565

[B7] Anzai K. Ogawa K. Kuniyasu A. Ozawa T. Yamamoto H. Nakayama H. (1998). Effects of hydroxyl radical and sulfhydryl reagents on the open probability of the purified cardiac ryanodine receptor channel incorporated into planar lipid bilayers. *Biochem. Biophys. Res. Commun.* 249 938–942. 10.1006/bbrc.1998.9244 9731240

[B8] Aon M. A. Cortassa S. Marbán E. O’Rourke B. (2003). Synchronized whole cell oscillations in mitochondrial metabolism triggered by a local release of reactive oxygen species in cardiac myocytes. *J. Biol. Chem.* 278 44735–44744. 10.1074/jbc.m302673200 12930841

[B9] Argaud L. Cour M. Dubien P. Y. Giraud F. Jossan C. Riche B. (2016). Effect of cyclosporine in nonshockable out-of-hospital cardiac arrest: the CYRUS randomized clinical trial. *JAMA Cardiol.* 1 557–565. 10.1001/jamacardio.2016.1701 27433815

[B10] Arteaga D. Odor A. López R. M. Contreras G. Pichardo J. García E. (1992). Impairment by cyclosporin A of reperfusion-induced arrhythmias. *Life Sci.* 51 1127–1134. 10.1016/0024-3205(92)90514-p 1518376

[B11] Askari G. Ghiasvand R. Feizi A. Ghanadian S. M. Karimian J. (2012). The effect of quercetin supplementation on selected markers of inflammation and oxidative stress. *J. Res. Med. Sci.* 17 637–641. 23798923 PMC3685779

[B12] Avula U. M. R. Hernandez J. J. Yamazaki M. Valdivia C. R. Chu A. Rojas-Pena A. (2018). Atrial infarction-induced spontaneous focal discharges and atrial fibrillation in sheep: role of dantrolene-sensitive aberrant ryanodine receptor calcium release. *Circ. Arrhythm. Electrophysiol.* 11:e005659. 10.1161/CIRCEP.117.005659 29540372 PMC6554725

[B13] Ayoub I. M. Radhakrishnan J. Gazmuri R. J. (2017). *In vivo* opening of the mitochondrial permeability transition pore in a rat model of ventricular fibrillation and closed-chest resuscitation. *Am. J. Transl. Res.* 9 3345–3359. 28804552 PMC5553884

[B14] Bannister M. L. Thomas N. L. Sikkel M. B. Mukherjee S. Maxwell C. MacLeod K. T. (2015). The mechanism of flecainide action in CPVT does not involve a direct effect on RyR2. *Circ. Res.* 116 1324–1335. 10.1161/circresaha.116.305347 25648700

[B15] Bassani J. W. Bassani R. A. Bers D. M. (1994). Relaxation in rabbit and rat cardiac cells: species-dependent differences in cellular mechanisms. *J. Physiol.* 476 279–293. 10.1113/jphysiol.1994.sp020130 8046643 PMC1160440

[B16] Belevych A. E. Terentyev D. Terentyeva R. Ho H. T. Gyorke I. Bonilla I. M. (2012). Shortened Ca2+ signaling refractoriness underlies cellular arrhythmogenesis in a postinfarction model of sudden cardiac death. *Circ. Res.* 110 569–577. 10.1161/circresaha.111.260455 22223353 PMC4068617

[B17] Bellinger A. M. Reiken S. Dura M. Murphy P. W. Deng S. X. Landry D. W. (2008). Remodeling of ryanodine receptor complex causes “leaky” channels: a molecular mechanism for decreased exercise capacity. *Proc. Natl. Acad. Sci. U.S.A.* 105 2198–2202. 10.1073/pnas.0711074105 18268335 PMC2538898

[B18] Bers D. M. (2002). Cardiac excitation-contraction coupling. *Nature* 415 198–205. 11805843 10.1038/415198a

[B19] Beuckelmann D. J. Erdmann E. (1992). Ca(2+)-currents and intracellular [Ca2+]i-transients in single ventricular myocytes isolated from terminally failing human myocardium. *Basic Res. Cardiol.* 87(Suppl. 1) 235–243. 10.1007/978-3-642-72474-9_19 1497571

[B20] Beutner G. Sharma V. K. Giovannucci D. R. Yule D. I. Sheu S. S. (2001). Identification of a ryanodine receptor in rat heart mitochondria. *J. Biol. Chem.* 276 21482–21488. 10.1074/jbc.m101486200 11297554

[B21] Boengler K. Stahlhofen S. van de Sand A. Gres P. Ruiz-Meana M. Garcia-Dorado D. (2009). Presence of connexin 43 in subsarcolemmal, but not in interfibrillar cardiomyocyte mitochondria. *Basic Res. Cardiol.* 104 141–147. 10.1007/s00395-009-0007-5 19242638

[B22] Böhm A. Tothova L. Urban L. Slezak P. Bacharova L. Musil P. (2016). The relation between oxidative stress biomarkers and atrial fibrillation after pulmonary veins isolation. *J. Electrocardiol.* 49 423–428. 10.1016/j.jelectrocard.2016.03.007 27034122

[B23] Boraso A. Williams A. J. (1994). Modification of the gating of the cardiac sarcoplasmic reticulum Ca(2+)-release channel by H_2_O_2_ and dithiothreitol. *Am. J. Physiol.* 267(3 Pt 2), H1010–H1016. 8092267 10.1152/ajpheart.1994.267.3.H1010

[B24] Bovo E. Lipsius S. L. Zima A. V. (2012). Reactive oxygen species contribute to the development of arrhythmogenic Ca^2+^ waves during β-adrenergic receptor stimulation in rabbit cardiomyocytes. *J. Physiol.* 590 3291–3304. 10.1113/jphysiol.2012.230748 22586224 PMC3459043

[B25] Brown D. A. Aon M. A. Akar F. G. Liu T. Sorarrain N. O’Rourke B. (2008). Effects of 4’-chlorodiazepam on cellular excitation–contraction coupling and ischaemia–reperfusion injury in rabbit heart. *Cardiovasc. Res.* 79 141–149. 10.1093/cvr/cvn053 18304929 PMC2562874

[B26] Bugger H. Abel E. D. (2014). Molecular mechanisms of diabetic cardiomyopathy. *Diabetologia* 57 660–671. 10.1007/s00125-014-3171-6 24477973 PMC3969857

[B27] Cerrone M. van Opbergen C. J. M. Malkani K. Irrera N. Zhang M. Van Veen T. A. B. (2018). Blockade of the adenosine 2A receptor mitigates the cardiomyopathy induced by loss of Plakophilin-2 expression. *Front. Physiol.* 9:1750. 10.3389/fphys.2018.01750 30568602 PMC6290386

[B28] Chelu M. G. Sarma S. Sood S. Wang S. van Oort R. J. Skapura D. G. (2009). Calmodulin kinase II–mediated sarcoplasmic reticulum Ca^2+^ leak promotes atrial fibrillation in mice. *J. Clin. Invest.* 119 1940–1951. 10.1172/jci37059 19603549 PMC2701862

[B29] Cheng H. Lederer W. J. (2008). Calcium sparks. *Physiol. Rev.* 88 1491–1545. 18923188 10.1152/physrev.00030.2007

[B30] Cho J. Won K. Wu D. Soong Y. Liu S. Szeto H. H. (2007). Potent mitochondria-targeted peptides reduce myocardial infarction in rats. *Coron. Artery Dis.* 18 215–220. 10.1097/01.mca.0000236285.71683.b6 17429296

[B31] Chu G. Luo W. Slack J. P. Tilgmann C. Sweet W. E. Spindler M. (1996). Compensatory mechanisms associated with the hyperdynamic function of phospholamban-deficient mouse hearts. *Circ. Res.* 79 1064–1076. 10.1161/01.res.79.6.1064 8943945

[B32] Cooper L. L. Li W. Lu Y. Centracchio J. Terentyeva R. Koren G. (2013). Redox modification of ryanodine receptors by mitochondria-derived reactive oxygen species contributes to aberrant Ca2+ handling in ageing rabbit hearts. *J. Physiol.* 591 5895–5911. 10.1113/jphysiol.2013.260521 24042501 PMC3872760

[B33] Crompton M. (1990). “The role of Ca2+ in the function and dysfunction of heart mitochondria,” in *Calcium and the Heart*, ed. Langer G. A., (New York, NY: Raven Press), 167–198.

[B34] Crompton M. Ellinger H. Costi A. (1988). Inhibition by cyclosporin A of a Ca2+-dependent pore in heart mitochondria activated by inorganic phosphate and oxidative stress. *Biochem. J.* 255 357–360. 3196322 PMC1135230

[B35] Csordás G. Renken C. Várnai P. Walter L. Weaver D. Buttle K. F. (2006). Structural and functional features and significance of the physical linkage between ER and mitochondria. *J. Cell Biol.* 174 915–921. 10.1083/jcb.200604016 16982799 PMC2064383

[B36] Davidson A. M. Halestrap A. P. (1990). Partial inhibition by cyclosporin A of the swelling of liver mitochondria in vivo and in vitro induced by sub-micromolar [Ca2+], but not by butyrate. Evidence for two distinct swelling mechanisms. *Biochem. J.* 268 147–152. 10.1042/bj2680147 2344354 PMC1131404

[B37] de Brito O. M. Scorrano L. (2008). Mitofusin 2 tethers endoplasmic reticulum to mitochondria. *Nature* 456 605–610. 10.1038/nature07534 19052620

[B38] De Vos K. J. Mórotz G. M. Stoica R. Tudor E. L. Lau K. F. Ackerley S. (2012). VAPB interacts with the mitochondrial protein PTPIP51 to regulate calcium homeostasis. *Hum. Mol. Genet.* 21 1299–1311. 10.1093/hmg/ddr559 22131369 PMC3284118

[B39] Deng Y. Zhang Z. S. (1993). Protective effects of combination of chlorpromazine and verapamil on ischemia-reperfusion induced injury in rat myocardium. *Yao Xue Xue Bao* 28 561–566. 8285062

[B40] Dey S. DeMazumder D. Sidor A. Foster D. B. O’Rourke B. (2018). Mitochondrial ROS drive sudden cardiac death and chronic proteome remodeling in heart failure. *Circ. Res.* 123 356–371. 10.1161/circresaha.118.312708 29898892 PMC6057154

[B41] Di Pasquale E. Lodola F. Miragoli M. Denegri M. Avelino-Cruz J. E Buonocore M. (2013). CaMKII inhibition rectifies arrhythmic phenotype in a patient-specific model of catecholaminergic polymorphic ventricular tachycardia. *Cell Death Dis.* 4:e843. 10.1038/cddis.2013.369 24113177 PMC3824678

[B42] Dolmatova E. Spagnol G. Boassa D. Baum J. R. Keith K. Ambrosi C. (2012). Cardiomyocyte ATP release through pannexin 1 aids in early fibroblast activation. *Am. J. Physiol. Heart Circ. Physiol.* 303 H1208–H1218. 22982782 10.1152/ajpheart.00251.2012 PMC3517637

[B43] Dong Z. Shanmughapriya S. Tomar D. Siddiqui N. Lynch S. Nemani N. (2017). Mitochondrial Ca(2+) uniporter is a mitochondrial luminal redox sensor that augments MCU channel activity. *Mol. Cell* 65 1014–1028.e7. 28262504 10.1016/j.molcel.2017.01.032 PMC5357178

[B44] Dries E. Santiago D. J. Gilbert G. Lenaerts I. Vandenberk B. Nagaraju C. K. (2018). Hyperactive ryanodine receptors in human heart failure and ischaemic cardiomyopathy reside outside of couplons. *Cardiovasc. Res.* 114 1512–1524. 10.1093/cvr/cvy088 29668881 PMC6106102

[B45] Eijsbouts S. C. Majidi M. van Zandvoort M. Allessie M. A. (2003). Effects of acute atrial dilation on heterogeneity in conduction in the isolated rabbit heart. *J. Cardiovasc. Electrophysiol.* 14 269–278. 10.1046/j.1540-8167.2003.02280.x 12716109

[B46] Eisner D. A. Caldwell J. L. Kistamas K. Trafford A. W. (2017). Calcium and excitation-contraction coupling in the heart. *Circ. Res.* 121 181–195. 10.1016/b978-012436570-4/50007-8 28684623 PMC5497788

[B47] Elgebaly S. A. Allam M. E. Houser S. Hashmi F. Forouhar F. Miano D. (1993). Cyclocreatine inhibits neutrophil accumulation in the myocardium of a canine model of coronary artery occlusion and reperfusion. *J. Pharmacol. Exp. Ther.* 266 1670–1677. 8371165

[B48] Elgebaly S. A. Allam M. E. Rossomando E. F. Cordis G. A. Forouhar F. Farghaly A. (1990). Cyclocreatine inhibits the production of neutrophil chemotactic factors from isolated hearts. *Am. J. Pathol.* 137 1233–1241. 2240167 PMC1877674

[B49] Elgebaly S. A. Wei Z. Tyles E. Elkerm A. F. Houser S. L. Gillies C. (1994). Enhancement of the recovery of rat hearts after prolonged cold storage by cyclocreatine phosphate. *Transplantation* 57 803–806. 10.1097/00007890-199403270-00005 8154024

[B50] El-Hamamsy I. Stevens L. M. Carrier M. Pellerin M. Bouchard D. Demers P. (2007). Effect of intravenous N-acetylcysteine on outcomes after coronary artery bypass surgery: a randomized, double-blind, placebo-controlled clinical trial. *J. Thorac. Cardiovasc. Surg.* 133 7–12. 10.1016/j.jtcvs.2006.05.070 17198774

[B51] Erickson J. R. Joiner M. L. Guan X. Kutschke W. Yang J. Oddis C. V. (2008). A dynamic pathway for calcium-independent activation of CaMKII by methionine oxidation. *Cell* 133 462–474. 10.1016/j.cell.2008.02.048 18455987 PMC2435269

[B52] Erickson J. R. Pereira L. Wang L. Han G. Ferguson A. Dao K. (2013). Diabetic hyperglycaemia activates CaMKII and arrhythmias by *O*-linked glycosylation. *Nature* 502 372–376. 10.1038/nature12537 24077098 PMC3801227

[B53] Fauconnier J. Thireau J. Reiken S. Cassan C. Richard S. Matecki S. (2010). Leaky RyR2 trigger ventricular arrhythmias in Duchenne muscular dystrophy. *Proc. Natl. Acad. Sci. U.S.A.* 107 1559–1564. 10.1073/pnas.0908540107 20080623 PMC2824377

[B54] Federico M. Valverde C. A. Mattiazzi A. Palomeque J. (2019). Unbalance between sarcoplasmic reticulum Ca^2+^ uptake and release: a first step toward Ca^2+^ triggered arrhythmias and cardiac damage. *Front. Physiol.* 10:1630. 10.3389/fphys.2019.01630 32038301 PMC6989610

[B55] Fernandez-Sada E. Silva-Platas C. Villegas C. A. Rivero S. L. Willis B. C. Garcia N. (2014). Cardiac responses to beta-adrenoceptor stimulation is partly dependent on mitochondrial calcium uniporter activity. *Br. J. Pharmacol.* 171 4207–4221. 10.1111/bph.12684 24628066 PMC4241088

[B56] Fernandez-Sanz C. Ruiz-Meana M. Miro-Casas E. Nuñez E. Castellano J. Loureiro M. (2014). Defective sarcoplasmic reticulum-mitochondria calcium exchange in aged mouse myocardium. *Cell Death Dis.* 5:e1573. 10.1038/cddis.2014.526 25522267 PMC4454162

[B57] Fill M. Copello J. A. (2002). Ryanodine receptor calcium release channels. *Physiol. Rev.* 82 893–922. 10.1016/s0006-3495(95)80265-1 12270947

[B58] Fischer E. Gottschalk A. Schüler C. (2017). An optogenetic arrhythmia model to study catecholaminergic polymorphic ventricular tachycardia mutations. *Sci. Rep.* 7:17514. 10.1038/s41598-017-17819-8 29235522 PMC5727474

[B59] Flores-Mateo G. Navas-Acien A. Pastor-Barriuso R. Guallar E. (2006). Selenium and coronary heart disease: a meta-analysis. *Am. J. Clin. Nutr.* 84 762–773. 10.1093/ajcn/84.4.762 17023702 PMC1829306

[B60] Formica J. V. Regelson W. (1995). Review of the biology of Quercetin and related bioflavonoids. *Food Chem. Toxicol.* 33 1061–1080. 10.1016/0278-6915(95)00077-1 8847003

[B61] Friedman J. R. Lackner L. L. West M. DiBenedetto J. R. Nunnari J. Voeltz G. K. (2011). ER tubules mark sites of mitochondrial division. *Science* 334 358–362. 10.1126/science.1207385 21885730 PMC3366560

[B62] Frommeyer G. Krawczyk J. Ellermann C. Bogeholz N. Kochhauser S. Dechering D. G. (2018). Ryanodine-receptor inhibition by dantrolene effectively suppresses ventricular arrhythmias in an *ex vivo* model of long-QT syndrome. *J. Cardiovasc. Electrophysiol.* 29 471–476. 10.1111/jce.13412 29314443

[B63] Gambardella J. Sorriento D. Ciccarelli M. Del Giudice C. Fiordelisi A. Napolitano L. (2017). Functional role of mitochondria in arrhythmogenesis. *Adv. Exp. Med. Biol.* 982 191–202. 10.1007/978-3-319-55330-6_10 28551788 PMC6709870

[B64] Garciarena C. D. Caldiz C. I. Correa M. V. Schinella G. R. Mosca S. M. Chiappe de Cingolani G. E. (2008). Na^+^/H^+^ exchanger-1 inhibitors decrease myocardial superoxide production via direct mitochondrial action. *J. Appl. Physiol.* 105 1706–1713. 10.1152/japplphysiol.90616.2008 18801963

[B65] García-Rivas G. D. J. Guerrero-Hernández A. Guerrero-Serna G. Rodríguez-Zavala J. S. Zazueta C. (2005). Inhibition of the mitochondrial calcium uniporter by the oxo-bridged dinuclear ruthenium amine complex (Ru360) prevents from irreversible injury in postischemic rat heart. *FEBS J.* 272 3477–3488. 10.1111/j.1742-4658.2005.04771.x 15978050

[B66] Garcia-Rivas Gde J. Carvajal K. Correa F. Zazueta C. (2006). Ru_360_, a specific mitochondrial calcium uptake inhibitor, improves cardiac post-ischaemic functional recovery in rats *in vivo*. *Br. J. Pharmacol.* 149 829–837. 10.1038/sj.bjp.0706932 17031386 PMC2014682

[B67] Giorgi C. Marchi S. Pinton P. (2018). The machineries, regulation and cellular functions of mitochondrial calcium. *Nat. Rev. Mol. Cell Biol.* 19 713–730. 10.1038/s41580-018-0052-8 30143745

[B68] Glancy B. Balaban R. S. (2012). Role of mitochondrial Ca2+ in the regulation of cellular energetics. *Biochemistry* 51 2959–2973. 10.1021/bi2018909 22443365 PMC3332087

[B69] Gonca E. Rapposelli S. Darıcı F. Digiacomo M. Yılmaz Z. (2016). Antiarrhythmic activity of a new spiro-cyclic benzopyran activator of the cardiac mitochondrial ATP dependent potassium channels. *Arch. Pharm. Res.* 39 1212–1222. 10.1007/s12272-016-0779-8 27357534

[B70] Gordan R. Fefelova N. Gwathmey J. K. Xie L. H. (2016). Involvement of mitochondrial permeability transition pore (mPTP) in cardiac arrhythmias: Evidence from cyclophilin D knockout mice. *Cell Calcium* 60 363–372. 10.1016/j.ceca.2016.09.001 27616659 PMC5127715

[B71] Gordan R. Fefelova N. Gwathmey J. K. Xie L. H. (2020). Iron overload, oxidative stress and calcium mishandling in Cardiomyocytes: role of the mitochondrial permeability transition pore. *Antioxidants* 9:758. 10.3390/antiox9080758 32824344 PMC7465659

[B72] Greenberg B. Butler J. Felker G. M. Ponikowski P. Voors A. A. Desai A. S. (2016). Calcium upregulation by percutaneous administration of gene therapy in patients with cardiac disease (CUPID 2): a randomised, multinational, double-blind, placebo-controlled, phase 2b trial. *Lancet* 387 1178–1186. 10.1016/s0140-6736(16)00082-9 26803443

[B73] Griffiths E. J. Halestrap A. P. (1991). Further evidence that cyclosporin A protects mitochondria from calcium overload by inhibiting a matrix peptidyl-prolyl cis-trans isomerase. Implications for the immunosuppressive and toxic effects of cyclosporin. *Biochem. J.* 274(Pt 2) 611–614. 10.1042/bj2740611 1706598 PMC1150183

[B74] Hamilton S. Terentyeva R. Kim T. Y. Bronk P. Clements R. T. O-Uchi J. (2018). Pharmacological modulation of mitochondrial Ca^2+^ content regulates sarcoplasmic reticulum Ca^2+^ release via oxidation of the ryanodine receptor by mitochondria-derived reactive oxygen species. *Front. Physiol.* 9:1831. 10.3389/fphys.2018.01831 30622478 PMC6308295

[B75] Hamilton S. Terentyeva R. Martin B. Perger F. Li J. Stepanov A. (2020). Increased RyR2 activity is exacerbated by calcium leak-induced mitochondrial ROS. *Basic Res. Cardiol.* 115:38. 10.1007/s00395-020-0797-z 32444920 PMC7244455

[B76] Harada M. Nattel S. N. Nattel S. (2012). AMP-activated protein kinase: potential role in cardiac electrophysiology and arrhythmias. *Circ. Arrhythm. Electrophysiol.* 5 860–867. 10.1161/circep.112.972265 22895602

[B77] Harman D. (1956). Aging: a theory based on free radical and radiation chemistry. *J. Gerontol.* 11 298–300. 10.1093/geronj/11.3.298 13332224

[B78] Harris D. A. Das A. M. (1991). Control of mitochondrial ATP synthesis in the heart. *Biochem. J.* 280(Pt 3) 561–573. 10.1042/bj2800561 1837214 PMC1130493

[B79] Hartmann N. Pabel S. Herting J. Schatter F. Renner A. Gummert J. (2017). Antiarrhythmic effects of dantrolene in human diseased cardiomyocytes. *Heart Rhythm.* 14 412–419. 10.1016/j.hrthm.2016.09.014 27650424

[B80] Hernández-Esquivel L. Pavón N. Buelna-Chontal M. González-Pacheco H. Belmont J. Chávez E. (2014). Citicoline (CDP-choline) protects myocardium from ischemia/reperfusion injury via inhibiting mitochondrial permeability transition. *Life Sci.* 96 53–58. 10.1016/j.lfs.2013.12.026 24389400

[B81] Herraiz-Martínez A. Álvarez-García J. Llach A. Molina C. E. Fernandes J. Ferrero-Gregori A. (2015). Ageing is associated with deterioration of calcium homeostasis in isolated human right atrial myocytes. *Cardiovasc. Res.* 106 76–86. 10.1093/cvr/cvv046 25712961 PMC4362404

[B82] Hicks J. J. Montes-Cortes D. H. Cruz-Dominguez M. P. Medina-Santillan R. Olivares-Corichi I. M. (2007). Antioxidants decrease reperfusion induced arrhythmias in myocardial infarction with ST-elevation. *Front. Biosci.* 12 2029–2037. 10.2741/2208 17127441

[B83] Hobai I. A. Buys E. S. Morse J. C. Edgecomb J. Weiss E. H. Armoundas A. A. (2013). SERCA Cys674 sulphonylation and inhibition of L-type Ca2+ influx contribute to cardiac dysfunction in endotoxemic mice, independent of cGMP synthesis. *Am. J. Physiol. Heart Circ. Physiol.* 305 H1189–H1200. 23934853 10.1152/ajpheart.00392.2012 PMC3798783

[B84] Horjus D. L. Oudman I. van Montfrans G. A. Brewster L. M. (2011). Creatine and creatine analogues in hypertension and cardiovascular disease. *Cochrane Database Syst. Rev.* 2011:Cd005184. 10.1002/14651858.CD005184.pub2 PMC6823205 22071819

[B85] Houser S. L. Elkerm A. F. Wei Z. Doyle K. Houser D. Liu X. K. (1995). Enhancement of cardiac function by cyclocreatine in models of cardiopulmonary bypass. *J. Mol. Cell Cardiol.* 27 1065–1073. 10.1016/0022-2828(95)90075-6 7563103

[B86] Hung L. M. Chen J. K. Huang S. S. Lee R. S. Su M. J. (2000). Cardioprotective effect of resveratrol, a natural antioxidant derived from grapes. *Cardiovasc. Res.* 47 549–555. 10.1016/s0008-6363(00)00102-4 10963727

[B87] Ito K. Shigematsu S. Sato T. Abe T. Li Y. Arita M. (2000). JTV-519, a novel cardioprotective agent, improves the contractile recovery after ischaemia-reperfusion in coronary perfused guinea-pig ventricular muscles. *Br. J. Pharmacol.* 130 767–776. 10.1038/sj.bjp.0703373 10864882 PMC1572131

[B88] Jansen J. A. van Veen T. A. de Bakker J. M. van Rijen H. V. (2010). Cardiac connexins and impulse propagation. *J. Mol. Cell Cardiol.* 48 76–82. 10.1016/j.yjmcc.2009.08.018 19729017

[B89] Jaski B. E. Jessup M. L. Mancini D. M. Cappola T. P. Pauly D. F. Greenberg B. (2009). Calcium upregulation by percutaneous administration of gene therapy in cardiac disease (CUPID Trial), a first-in-human phase 1/2 clinical trial. *J. Card Fail.* 15 171–181. 10.1016/j.cardfail.2009.01.013 19327618 PMC2752875

[B90] Jeong E. M. Liu M. Sturdy M. Gao G. Varghese S. T. Sovari A. A. (2012). Metabolic stress, reactive oxygen species, and arrhythmia. *J. Mol. Cell Cardiol.* 52 454–463. 21978629 10.1016/j.yjmcc.2011.09.018 PMC3264827

[B91] Jessup M. Greenberg B. Mancini D. Cappola T. Pauly D. F. Jaski B. (2011). Calcium upregulation by percutaneous administration of Gene Therapy in Cardiac Disease (CUPID): a phase 2 trial of intracoronary gene therapy of sarcoplasmic reticulum Ca2+-ATPase in patients with advanced heart failure. *Circulation* 124 304–313. 10.1161/circulationaha.111.022889 21709064 PMC5843948

[B92] Jin J. K. Blackwood E. A. Azizi K. Thuerauf D. J. Fahem A. G. Hofmann C. (2017). ATF6 decreases myocardial ischemia/reperfusion damage and links ER Stress and oxidative stress signaling pathways in the heart. *Circ. Res.* 120 862–875. 10.1161/circresaha.116.310266 27932512 PMC5336510

[B93] Joiner M. L. Koval O. M. (2014). CaMKII and stress mix it up in mitochondria. *Front. Pharmacol.* 5:67. 10.3389/fphar.2014.00067 24822046 PMC4013469

[B94] Joiner M. L. Koval O. M. Li J. He B. J. Allamargot C. Gao Z. (2012). CaMKII determines mitochondrial stress responses in heart. *Nature* 491 269–273. 10.1038/nature11444 23051746 PMC3471377

[B95] Kazak L. Chouchani E. T. Stavrovskaya I. G. Lu G. Z. Jedrychowski M. P. Egan D. F. (2017). UCP1 deficiency causes brown fat respiratory chain depletion and sensitizes mitochondria to calcium overload-induced dysfunction. *Proc. Natl. Acad. Sci. U.S.A.* 114 7981–7986. 10.1073/pnas.1705406114 28630339 PMC5544316

[B96] Khoo M. S. Li J. Singh M. V. Yang Y. Kannankeril P. Wu Y. (2006). Death, cardiac dysfunction, and arrhythmias are increased by calmodulin kinase II in calcineurin cardiomyopathy. *Circulation* 114 1352–1359. 10.1161/circulationaha.106.644583 16982937

[B97] Khurshid S. Choi S. H. Weng L. C. Wang E. Y. Trinquart L. Benjamin E. J. (2018). Frequency of cardiac rhythm abnormalities in a half million adults. *Circ. Arrhythm. Electrophysiol.* 11:e006273. 10.1161/circep.118.006273 29954742 PMC6051725

[B98] Kim J. C. Pérez-Hernández M. Alvarado F. J. Maurya S. R. Montnach J. Yin Y. (2019). Disruption of Ca(2+)(i) homeostasis and connexin 43 hemichannel function in the right ventricle precedes overt arrhythmogenic cardiomyopathy in Plakophilin-2-deficient mice. *Circulation* 140 1015–1030. 10.1161/circulationaha.119.039710 31315456 PMC6746608

[B99] Kim Y. H. Lim D. S. Lee J. H. Shim W. J. Ro Y. M. Park G. H. (2003). Gene expression profiling of oxidative stress on atrial fibrillation in humans. *Exp. Mol. Med.* 35 336–349. 10.1038/emm.2003.45 14646586

[B100] Kimura H. Kawahara K. Yamauchi Y. Miyaki J. (2005). On the mechanisms for the conversion of ventricular fibrillation to tachycardia by perfusion with ruthenium red. *J. Electrocardiol.* 38 364–370. 10.1016/j.jelectrocard.2005.05.007 16216614

[B101] Kirchhefer U. Schmitz W. Scholz H. Neumann J. (1999). Activity of cAMP-dependent protein kinase and Ca2+/calmodulin-dependent protein kinase in failing and nonfailing human hearts. *Cardiovasc. Res.* 42 254–261. 10.1016/s0008-6363(98)00296-x 10435018

[B102] Kirchhoff S. Nelles E. Hagendorff A. Krüger O. Traub O. Willecke K. (1998). Reduced cardiac conduction velocity and predisposition to arrhythmias in connexin40-deficient mice. *Curr. Biol.* 8 299–302. 10.1016/s0960-9822(98)70114-9 9501070

[B103] Knottnerus S. J. G. Mengarelli I. Wüst R. C. I. Baartscheer A. Bleeker J. C. Coronel R. (2020). Electrophysiological abnormalities in VLCAD Deficient hiPSC-Cardiomyocytes can be improved by lowering accumulation of fatty acid oxidation intermediates. *Int. J. Mol. Sci.* 21:2589. 10.3390/ijms21072589 32276429 PMC7177397

[B104] Kobayashi S. Bannister M. L. Gangopadhyay J. P. Hamada T. Parness J. Ikemoto N. (2005). Dantrolene stabilizes domain interactions within the ryanodine receptor. *J. Biol. Chem.* 280 6580–6587. 10.1074/jbc.m408375200 15611117

[B105] Kobayashi S. Yano M. Uchinoumi H. Suetomi T. Susa T. Ono M. (2010). Dantrolene, a therapeutic agent for malignant hyperthermia, inhibits catecholaminergic polymorphic ventricular tachycardia in a RyR2^*R2474S*/+^ knock-in mouse model. *Circ. J.* 74 2579–2584. 10.1253/circj.cj-10-0680 20944434

[B106] Kohlhaas M. Maack C. (2013). Calcium release microdomains and mitochondria. *Cardiovasc. Res.* 98 259–268. 10.1093/cvr/cvt032 23417042

[B107] Kohno M. Yano M. Kobayashi S. Doi M. Oda T. Tokuhisa T. (2003). A new cardioprotective agent, JTV519, improves defective channel gating of ryanodine receptor in heart failure. *Am. J. Physiol. Heart Circ. Physiol.* 284 H1035–H1042. 10.1152/ajpheart.00722.2002 12433661

[B108] Kónya L. Kékesi V. Juhász-Nagy S. Fehér J. (1992). The effect of superoxide dismutase in the myocardium during reperfusion in the dog. *Free Radic. Biol. Med.* 13 527–532. 10.1016/0891-5849(92)90147-9 1281132

[B109] Korobova F. Ramabhadran V. Higgs H. N. (2013). An actin-dependent step in mitochondrial fission mediated by the ER-associated formin INF2. *Science* 339 464–467. 10.1126/science.1228360 23349293 PMC3843506

[B110] Krul S. P. Berger W. R. Smit N. W. van Amersfoorth S. C. Driessen A. H. van Boven W. J. (2015). Atrial fibrosis and conduction slowing in the left atrial appendage of patients undergoing thoracoscopic surgical pulmonary vein isolation for atrial fibrillation. *Circ. Arrhythm. Electrophysiol.* 8 288–295. 10.1161/circep.114.001752 25673630

[B111] Kryshtal D. O. Blackwell D. Egly C. Smith A. N. Batiste S. M. Johnston J. N. (2020). RYR2 channel inhibition is the principal mechanism 0f Flecainide Action in CPVT. *Circ Res.* 10.1161/CIRCRESAHA.120.316819 [Epub ahead of print]. 33297863 PMC7864884

[B112] Kushnir A. Shan J. Betzenhauser M. J. Reiken S. Marks A. R. (2010). Role of CaMKIIdelta phosphorylation of the cardiac ryanodine receptor in the force frequency relationship and heart failure. *Proc. Natl. Acad. Sci. U.S.A.* 107 10274–10279. 10.1073/pnas.1005843107 20479242 PMC2890457

[B113] Kwong J. Q. Lu X. Correll R. N. Schwanekamp J. A. Vagnozzi R. J. Sargent M. A. (2015). The mitochondrial calcium uniporter selectively matches metabolic output to acute contractile stress in the heart. *Cell Rep.* 12 15–22. 10.1016/j.celrep.2015.06.002 26119742 PMC4497842

[B114] Langer G. A. Peskoff A. (1996). Calcium concentration and movement in the diadic cleft space of the cardiac ventricular cell. *Biophys. J.* 70 1169–1182. 10.1016/s0006-3495(96)79677-7 8785276 PMC1225046

[B115] Lara-Vaca S. Cordero-Cabra A. Martínez-Flores E. Iturralde-Torres P. (2014). Registro mexicano de fibrilación auricular (ReMeFa). *Gaceta Méd. México* 150 48–59. 25643677

[B116] Larbig R. Reda S. Paar V. Trost A. Leitner J. Weichselbaumer S. (2017). Through modulation of cardiac Ca(2+) handling, UCP2 affects cardiac electrophysiology and influences the susceptibility for Ca(2+) -mediated arrhythmias. *Exp. Physiol.* 102 650–662. 10.1113/ep086209 28370799

[B117] Leducq N. Bono F. Sulpice T. Vin V. Janiak P. Fur G. L. (2003). Role of peripheral benzodiazepine receptors in mitochondrial, cellular, and cardiac damage induced by oxidative stress and ischemia-reperfusion. *J. Pharmacol. Exp. Ther.* 306 828–837. 10.1124/jpet.103.052068 12928523

[B118] Lee D. S. Gona P. Albano I. Larson M. G. Benjamin E. J. Levy D. (2011). A systematic assessment of causes of death after heart failure onset in the community: impact of age at death, time period, and left ventricular systolic dysfunction. *Circ. Heart Fail.* 4 36–43. 10.1161/circheartfailure.110.957480 21071547 PMC3243964

[B119] Lehnart S. E. Mongillo M. Bellinger A. Lindegger N. Chen B. X. Hsueh W. (2008). Leaky Ca^2+^ release channel/ryanodine receptor 2 causes seizures and sudden cardiac death in mice. *J. Clin. Invest.* 118 2230–2245. 10.1172/JCI35346 18483626 PMC2381750

[B120] Lehnart S. E. Wehrens X. H. Laitinen P. J. Reiken S. R. Deng S. X. Cheng Z. (2004a). Sudden death in familial polymorphic ventricular tachycardia associated with calcium release channel (ryanodine receptor) leak. *Circulation* 109 3208–3214. 10.1161/01.cir.0000132472.98675.ec 15197150

[B121] Lehnart S. E. Wehrens X. H. Marks A. R. (2004b). Calstabin deficiency, ryanodine receptors, and sudden cardiac death. *Biochem. Biophys. Res. Commun.* 322 1267–1279. 10.1016/j.bbrc.2004.08.032 15336974

[B122] Lemme M. Braren I. Prondzynski M. Aksehirlioglu B. Ulmer B. M. Schulze M. L. (2019). Chronic intermittent tachypacing by an optogenetic approach induces arrhythmia vulnerability in human engineered heart tissue. *Cardiovasc. Res.* 116 1487–1499. 10.1093/cvr/cvz245 31598634 PMC7314638

[B123] Lin P. H. Lee S. H. Su C. P. Wei Y. H. (2003). Oxidative damage to mitochondrial DNA in atrial muscle of patients with atrial fibrillation. *Free Radic. Biol. Med.* 35 1310–1318. 10.1016/j.freeradbiomed.2003.07.002 14607530

[B124] Liu N. Ruan Y. Denegri M. Bachetti T. Li Y. Colombi B. (2011). Calmodulin kinase II inhibition prevents arrhythmias in RyR2^*R4496C*+/−^ mice with catecholaminergic polymorphic ventricular tachycardia. *J. Mol. Cell Cardiol.* 50 214–222. 10.1016/j.yjmcc.2010.10.001 20937285

[B125] Locovei S. Wang J. Dahl G. (2006). Activation of pannexin 1 channels by ATP through P2Y receptors and by cytoplasmic calcium. *FEBS Lett.* 580 239–244. 10.1016/j.febslet.2005.12.004 16364313

[B126] Lopez-Crisosto C. Pennanen C. Vasquez-Trincado C. Morales P. E. Bravo-Sagua R. Quest A. F. G. (2017). Sarcoplasmic reticulum-mitochondria communication in cardiovascular pathophysiology. *Nat. Rev. Cardiol.* 14 342–360. 10.1038/nrcardio.2017.23 28275246

[B127] Lozano O. Lázaro-Alfaro A. Silva-Platas C. Oropeza-Almazán Y. Torres-Quintanilla A. Bernal-Ramírez J. (2019). Nanoencapsulated quercetin improves cardioprotection during hypoxia-reoxygenation injury through preservation of mitochondrial function. *Oxid. Med. Cell Longev.* 2019:7683051. 10.1155/2019/7683051 31341535 PMC6612997

[B128] Lozano O. Torres-Quintanilla A. García-Rivas G. (2018). Nanomedicine for the cardiac myocyte: Where are we? *J. Control Release* 271 149–165. 10.1016/j.jconrel.2017.12.018 29273321

[B129] Lu X. Ginsburg K. S. Kettlewell S. Bossuyt J. Smith G. L. Bers D. M. (2013). Measuring local gradients of intramitochondrial [Ca(2+)] in cardiac myocytes during sarcoplasmic reticulum Ca(2+) release. *Circ. Res.* 112 424–431. 10.1161/circresaha.111.300501 23243207 PMC3566246

[B130] Maack C. O’Rourke B. (2007). Excitation-contraction coupling and mitochondrial energetics. *Basic Res. Cardiol.* 102 369–392. 10.1007/s00395-007-0666-z 17657400 PMC2785083

[B131] MacDonnell S. M. García-Rivas G. Scherman J. A. Kubo H. Chen X. Valdivia H. (2008). Adrenergic regulation of cardiac contractility does not involve phosphorylation of the cardiac ryanodine receptor at serine 2808. *Circ. Res.* 102 e65–e72. 18388322 10.1161/CIRCRESAHA.108.174722 PMC2652487

[B132] Maneechote C. Palee S. Kerdphoo S. Jaiwongkam T. Chattipakorn S. C. Chattipakorn N. (2018). Differential temporal inhibition of mitochondrial fission by Mdivi-1 exerts effective cardioprotection in cardiac ischemia/reperfusion injury. *Clin. Sci.* 132 1669–1683. 10.1042/cs20180510 30065084

[B133] Maneechote C. Palee S. Kerdphoo S. Jaiwongkam T. Chattipakorn S. C. Chattipakorn N. (2019). Balancing mitochondrial dynamics via increasing mitochondrial fusion attenuates infarct size and left ventricular dysfunction in rats with cardiac ischemia/reperfusion injury. *Clin. Sci.* 133 497–513. 10.1042/cs20190014 30705107

[B134] Manor U. Bartholomew S. Golani G. Christenson E. Kozlov M. Higgs H. (2015). A mitochondria-anchored isoform of the actin-nucleating spire protein regulates mitochondrial division. *eLife* 4:e08828. 10.7554/eLife.08828 PMC4574297 26305500

[B135] Martínez-González M. Toledo E. Arós F. Fiol M. Corella D. Salas-Salvadó J. (2014). Extravirgin olive oil consumption reduces risk of atrial fibrillation: the PREDIMED (Prevención con Dieta Mediterránea) trial. *Circulation* 130 18–26. 10.1161/circulationaha.113.006921 24787471

[B136] Martinvalet D. (2018). The role of the mitochondria and the endoplasmic reticulum contact sites in the development of the immune responses. *Cell Death Dis.* 9:336. 10.1038/s41419-017-0237-7 PMC5832423 29491398

[B137] Matlib M. A. Zhou Z. Knight S. Ahmed S. Choi K. M. Krause-Bauer J. (1998). Oxygen-bridged dinuclear ruthenium amine complex specifically inhibits Ca2+ uptake into mitochondria in vitro and in situ in single cardiac myocytes. *J. Biol. Chem.* 273 10223–10231. 10.1074/jbc.273.17.10223 9553073

[B138] McCormack J. G. Denton R. M. (1990). The role of mitochondrial Ca2+ transport and matrix Ca2+ in signal transduction in mammalian tissues. *Biochim. Biophys. Acta* 1018 287–291. 10.1016/0005-2728(90)90269-a 2203475

[B139] McDonald C. Fraser J. Shekar K. Clarke A. Coombes J. Barnett A. (2016). Low preoperative selenium is associated with post-operative atrial fibrillation in patients having intermediate-risk coronary artery surgery. *Eur. J. Clin. Nutr.* 70 1138–1143. 10.1038/ejcn.2016.125 27406157

[B140] Meissner G. (2004). Molecular regulation of cardiac ryanodine receptor ion channel. *Cell Calcium* 35 621–628. 10.1016/j.ceca.2004.01.015 15110152

[B141] Menezes-Rodrigues F. S. Tavares J. G. P. Vasques E. R. Errante P. R. Araújo E. A. Pires-Oliveira M. (2020). Cardioprotective effects of pharmacological blockade of the mitochondrial calcium uniporter on myocardial ischemia-reperfusion injury. *Acta Cir. Bras.* 35:e202000306. 10.1590/s0102-865020200030000006 PMC7251977 32692797

[B142] Motloch L. J. Hu J. Akar F. G. (2015). The mitochondrial translocator protein and arrhythmogenesis in ischemic heart disease. *Oxid. Med. Cell Longev.* 2015:234104. 10.1155/2015/234104 PMC4397036 25918579

[B143] Murphy K. R. Baggett B. Cooper L. L. Lu Y. Ou J. Sedivy J. M. (2019). Enhancing autophagy diminishes aberrant Ca^(2+)^ homeostasis and arrhythmogenesis in aging rabbit hearts. *Front. Physiol.* 10:1277. 10.3389/fphys.2019.01277 31636573 PMC6787934

[B144] Negi S. Shukrullah I. Veledar E. Bloom H. L. Jones D. P. Dudley S. C. (2011). Statin therapy for the prevention of atrial fibrillation trial (SToP AF trial). *J. Cardiovasc. Electrophysiol.* 22 414–419. 10.1111/j.1540-8167.2010.01925.x 20946227 PMC3022954

[B145] Ni R. Cao T. Xiong S. Ma J. Fan G. C. Lacefield J. C. (2016). Therapeutic inhibition of mitochondrial reactive oxygen species with mito-TEMPO reduces diabetic cardiomyopathy. *Free Radic. Biol. Med.* 90 12–23. 10.1016/j.freeradbiomed.2015.11.013 26577173 PMC5066872

[B146] Nickel A. G. Kohlhaas M. Bertero E. Wilhelm D. Wagner M. Sequeira V. (2020). CaMKII does not control mitochondrial Ca(2+) uptake in cardiac myocytes. *J. Physiol.* 598 1361–1376. 10.1113/jp276766 30770570

[B147] Nikolaienko R. Bovo E. Zima A. V. (2018). Redox dependent modifications of ryanodine receptor: basic mechanisms and implications in heart diseases. *Front. Physiol.* 9:1775. 10.3389/fphys.2018.01775 30574097 PMC6291498

[B148] O’Neill S. C. Miller L. Hinch R. Eisner D. A. (2004). Interplay between SERCA and sarcolemmal Ca2+ efflux pathways controls spontaneous release of Ca2+ from the sarcoplasmic reticulum in rat ventricular myocytes. *J. Physiol.* 559(Pt 1) 121–128. 10.1113/jphysiol.2003.058917 15194743 PMC1665077

[B149] Oropeza-Almazan Y. Blatter L. A. (2020). Mitochondrial calcium uniporter complex activation protects against calcium Alternans in Atrial Myocytes. *Am. J. Physiol. Heart Circ. Physiol.* 319 H873–H881. 32857593 10.1152/ajpheart.00375.2020 PMC7654655

[B150] Oropeza-Almazan Y. Vazquez-Garza E. Chapoy-Villanueva H. Torre-Amione G. Garcia-Rivas G. (2017). Small interfering RNA targeting mitochondrial calcium uniporter improves cardiomyocyte cell viability in hypoxia/reoxygenation injury by reducing calcium overload. *Oxid. Med. Cell Longev.* 2017:5750897. 10.1155/2017/5750897 PMC5350333 28337252

[B151] O’Rourke B. Blatter L. A. (2009). Mitochondrial Ca2+ uptake: tortoise or hare? *J. Mol. Cell Cardiol.* 46 767–774. 10.1016/j.yjmcc.2008.12.011 19162034 PMC4005816

[B152] Osbakken M. Ito K. Zhang D. Ponomarenko I. Ivanics T. Jahngen E. G. (1992). Creatine and cyclocreatine effects on ischemic myocardium: 31P nuclear magnetic resonance evaluation of intact heart. *Cardiology* 80 184–195. 10.1159/000175002 1511465

[B153] Ozcan C. Palmeri M. Horvath T. L. Russell K. S. Russell R. R. III (2013). Role of uncoupling protein 3 in ischemia-reperfusion injury, arrhythmias, and preconditioning. *Am. J. Physiol. Heart Circ. Physiol.* 304 H1192–H1200. 23457013 10.1152/ajpheart.00592.2012 PMC3652089

[B154] Pain T. Yang X. M. Critz S. D. Yue Y. Nakano A. Liu G. S. (2000). Opening of mitochondrial K(ATP) channels triggers the preconditioned state by generating free radicals. *Circ. Res.* 87 460–466. 10.1161/01.res.87.6.460 10988237

[B155] Papadatos G. A. Wallerstein P. M. Head C. E. Ratcliff R. Brady P. A. Benndorf K. (2002). Slowed conduction and ventricular tachycardia after targeted disruption of the cardiac sodium channel gene Scn5a. *Proc. Natl. Acad. Sci. U.S.A.* 99 6210–6215. 10.1073/pnas.082121299 11972032 PMC122928

[B156] Parra E. Cruz D. García G. Zazueta C. Correa F. García N. (2005). Myocardial protective effect of octylguanidine against the damage induced by ischemia reperfusion in rat heart. *Mol. Cell Biochem.* 269 19–26. 10.1007/s11010-005-2989-0 15786713

[B157] Paul-Pletzer K. Yamamoto T. Bhat M. B. Ma J. Ikemoto N. Jimenez L. S. (2002). Identification of a dantrolene-binding sequence on the skeletal muscle ryanodine receptor. *J. Biol. Chem.* 277 34918–34923. 10.1074/jbc.m205487200 12167662

[B158] Pavón N. Aranda A. García N. Hernández-Esquivel L. Chávez E. (2009). In hyperthyroid rats octylguanidine protects the heart from reperfusion damage. *Endocrine* 35 158–165. 10.1007/s12020-008-9144-0 19169849

[B159] Penttinen K. Swan H. Vanninen S. Paavola J. Lahtinen A. M. Kontula K. (2015). Antiarrhythmic effects of dantrolene in patients with catecholaminergic polymorphic ventricular tachycardia and replication of the responses using iPSC models. *PLoS One* 10:e0125366. 10.1371/journal.pone.0125366 25955245 PMC4425399

[B160] Purohit A. Rokita A. G. Guan X. Chen B. Koval O. M. Voigt N. (2013). Oxidized Ca(2+)/calmodulin-dependent protein kinase II triggers atrial fibrillation. *Circulation* 128 1748–1757. 10.1161/circulationaha.113.003313 24030498 PMC3876034

[B161] Qin F. Siwik D. A. Lancel S. Zhang J. Kuster G. M. Luptak I. (2013). Hydrogen peroxide-mediated SERCA cysteine 674 oxidation contributes to impaired cardiac myocyte relaxation in senescent mouse heart. *J. Am. Heart Assoc.* 2:e000184. 10.1161/JAHA.113.000184 PMC3828801 23963753

[B162] Qin Y. Li A. Liu B. Jiang W. Gao M. Tian X. (2020). Mitochondrial fusion mediated by fusion promotion and fission inhibition directs adult mouse heart function toward a different direction. *FASEB J.* 34 663–675. 10.1096/fj.201901671r 31914595

[B163] Qiu Y. Bernier M. Hearse D. J. (1990). The influence of N-acetylcysteine on cardiac function and rhythm disorders during ischemia and reperfusion. *Cardioscience* 1 65–74. 1715201

[B164] Ramesh V. Sharma V. K. Sheu S. S. Franzini-Armstrong C. (1998). Structural proximity of mitochondria to calcium release units in rat ventricular myocardium may suggest a role in Ca2+ sequestration. *Ann. N.Y. Acad. Sci.* 853 341–344. 10.1111/j.1749-6632.1998.tb08295.x 10603975

[B165] Robinson P. Liu X. Sparrow A. Patel S. Zhang Y. H. Casadei B. (2018). Hypertrophic cardiomyopathy mutations increase myofilament Ca(2+) buffering, alter intracellular Ca(2+) handling, and stimulate Ca(2+)-dependent signaling. *J. Biol. Chem.* 293 10487–10499. 10.1074/jbc.ra118.002081 29760186 PMC6036197

[B166] Rocca C. Boukhzar L. Granieri M. C. Alsharif I. Mazza R. Lefranc B. (2018). A selenoprotein T-derived peptide protects the heart against ischaemia/reperfusion injury through inhibition of apoptosis and oxidative stress. *Acta Physiol.* 223:e13067. 10.1111/apha.13067 29575758

[B167] Rodrigo R. Korantzopoulos P. Cereceda M. Asenjo R. Zamorano J. Villalabeitia E. (2013). A randomized controlled trial to prevent post-operative atrial fibrillation by antioxidant reinforcement. *J. Am. Coll. Cardiol.* 62 1457–1465. 10.1016/j.jacc.2013.07.014 23916928

[B168] Rodríguez-Reyes H. Muñoz Gutiérrez M. Márquez M. F. Pozas Garza G. Asensio Lafuente E. Ortíz Galván F. (2015). Muerte súbita cardiaca. Estratificación de riesgo, prevención y tratamiento. *Archivos Cardiol. México* 85 329–336. 10.1016/j.acmx.2015.06.002 26253348

[B169] Roussel J. Thireau J. Brenner C. Saint N. Scheuermann V. Lacampagne A. (2015). Palmitoyl-carnitine increases RyR2 oxidation and sarcoplasmic reticulum Ca^2+^ leak in cardiomyocytes: role of adenine nucleotide translocase. *Biochim. Biophys. Acta* 1852 749–758. 10.1016/j.bbadis.2015.01.011 25619687

[B170] Rutledge C. Dudley S. (2013). Mitochondria and arrhythmias. *Expert Rev. Cardiovasc. Ther.* 11 799–801. 10.1586/14779072.2013.811969 23895021 PMC4211874

[B171] Sag C. M. Wadsack D. P. Khabbazzadeh S. Abesser M. Grefe C. Neumann K. (2009). Calcium/calmodulin-dependent protein kinase II contributes to cardiac arrhythmogenesis in heart failure. *Circ. Heart Fail.* 2 664–675. 10.1161/circheartfailure.109.865279 19919992 PMC2835502

[B172] Sano T. Sugiyama S. Taki K. Hanaki Y. Shimada Y. Ozawa T. (1990). Effects of antiarrhythmic agents classified as class III group on ischaemia-induced myocardial damage in canine hearts. *Br. J. Pharmacol.* 99 577–581. 10.1111/j.1476-5381.1990.tb12971.x 2331583 PMC1917329

[B173] Santana L. F. Kranias E. G. Lederer W. J. (1997). Calcium sparks and excitation-contraction coupling in phospholamban-deficient mouse ventricular myocytes. *J. Physiol.* 503(Pt 1) 21–29. 10.1111/j.1469-7793.1997.021bi.x 9288671 PMC1159883

[B174] Santulli G. Xie W. Reiken S. R. Marks A. R. (2015). Mitochondrial calcium overload is a key determinant in heart failure. *Proc. Natl. Acad. Sci. U.S.A.* 112 11389–11394. 10.1073/pnas.1513047112 26217001 PMC4568687

[B175] Sardu C. Santulli G. Guerra G. Trotta M. C. Santamaria M. Sacra C. (2020). Modulation of SERCA in patients with persistent atrial fibrillation treated by epicardial thoracoscopic ablation: the CAMAF study. *J. Clin. Med.* 9:544. 10.3390/jcm9020544 32079238 PMC7074346

[B176] Sasaki K. Makiyama T. Yoshida Y. Wuriyanghai Y. Kamakura T. Nishiuchi S. (2016). Patient-specific human induced pluripotent stem cell model assessed with electrical pacing validates S107 as a potential therapeutic agent for Catecholaminergic polymorphic ventricular tachycardia. *PLoS One* 11:e0164795. 10.1371/journal.pone.0164795 27764147 PMC5072719

[B177] Schaper J. Meiser E. Stammler G. (1985). Ultrastructural morphometric analysis of myocardium from dogs, rats, hamsters, mice, and from human hearts. *Circ. Res.* 56 377–391. 10.1161/01.res.56.3.377 3882260

[B178] Schmidt A. G. Gerst M. Zhai J. Carr A. N. Pater L. Kranias E. G. (2002). Evaluation of left ventricular diastolic function from spectral and color M-mode doppler in genetically altered mice. *J. Am. Soc. Echocardiogr.* 15(10 Pt 1), 1065–1073. 10.1067/mje.2002.121863 12373248

[B179] Schreiner K. D. Kelemen K. Zehelein J. Becker R. Senges J. C. Bauer A. (2004). Biventricular hypertrophy in dogs with chronic AV block: effects of cyclosporin A on morphology and electrophysiology. *Am. J. Physiol. Heart Circ. Physiol.* 287 H2891–H2898. 10.1152/ajpheart.01051.2003 15178548

[B180] Schweitzer M. K. Wilting F. Sedej S. Dreizehnter L. Dupper N. J. Tian Q. (2017). Suppression of arrhythmia by enhancing mitochondrial Ca^2+^ uptake in Catecholaminergic ventricular tachycardia models. *JACC Basic Transl. Sci.* 2 737–747. 10.1016/j.jacbts.2017.06.008 29354781 PMC5774336

[B181] Scriven D. R. Asghari P. Moore E. D. (2013). Microarchitecture of the dyad. *Cardiovasc. Res.* 98 169–176. 10.1093/cvr/cvt025 23400762

[B182] Shaikh G. Zhang J. Perez-Aso M. Mediero A. Cronstein B. (2016). Adenosine A(2A) receptor promotes collagen type III synthesis via β-catenin activation in human dermal fibroblasts. *Br. J. Pharmacol.* 173 3279–3291. 10.1111/bph.13615 27595240 PMC5738670

[B183] Shan J. Xie W. Betzenhauser M. Reiken S. Chen B. X. Wronska A. (2012). Calcium leak through ryanodine receptors leads to atrial fibrillation in 3 mouse models of catecholaminergic polymorphic ventricular tachycardia. *Circ. Res.* 111 708–717. 10.1161/circresaha.112.273342 22828895 PMC3734386

[B184] Sharma V. Abraham T. So A. Allard M. F. McNeill J. H. (2010). Functional effects of protein kinases and peroxynitrite on cardiac carnitine palmitoyltransferase-1 in isolated mitochondria. *Mol. Cell Biochem.* 337 223–237. 10.1007/s11010-009-0303-2 19862603

[B185] Sheu S. S. Lederer W. J. (1985). Lidocaine’s negative inotropic and antiarrhythmic actions. Dependence on shortening of action potential duration and reduction of intracellular sodium activity. *Circ. Res.* 57 578–590. 10.1161/01.res.57.4.578 2412723

[B186] Shimizu H. Schredelseker J. Huang J. Lu K. Naghdi S. Lu F. (2015). Mitochondrial Ca^2+^ uptake by the voltage-dependent anion channel 2 regulates cardiac rhythmicity. *eLife* 4:e04801. 10.7554/eLife.04801 25588501 PMC4293673

[B187] Smeets J. L. Allessie M. A. Lammers W. J. Bonke F. I. Hollen J. (1986). The wavelength of the cardiac impulse and reentrant arrhythmias in isolated rabbit atrium. The role of heart rate, autonomic transmitters, temperature, and potassium. *Circ. Res.* 58 96–108. 10.1161/01.res.58.1.96 3943157

[B188] Smith R. M. Visweswaran R. Talkachova I. Wothe J. K. Tolkacheva E. G. (2013). Uncoupling the mitochondria facilitates alternans formation in the isolated rabbit heart. *Am. J. Physiol. Heart Circ. Physiol.* 305 H9–H18. 23645464 10.1152/ajpheart.00915.2012

[B189] Sochman J. Kolc J. Vrána M. Fabián J. (1990). Cardioprotective effects of *N*-acetylcysteine: the reduction in the extent of infarction and occurrence of reperfusion arrhythmias in the dog. *Int. J. Cardiol.* 28 191–196. 10.1016/0167-5273(90)90060-i 2394523

[B190] Soloviev A. Stefanov A. Parshikov A. Khromov A. Moibenko A. Kvotchina L. (2002). Arrhythmogenic peroxynitrite-induced alterations in mammalian heart contractility and its prevention with quercetin-filled liposomes. *Cardiovasc. Toxicol.* 2 129–139. 10.1385/ct:2:2:129 12271156

[B191] Sood S. Chelu M. G. van Oort R. J. Skapura D. Santonastasi M. Dobrev D. (2008). Intracellular calcium leak due to FKBP12.6 deficiency in mice facilitates the inducibility of atrial fibrillation. *Heart Rhythm.* 5 1047–1054. 10.1016/j.hrthm.2008.03.030 18598963 PMC2525570

[B192] Sossalla S. Fluschnik N. Schotola H. Ort K. R. Neef S. Schulte T. (2010). Inhibition of elevated Ca^2+^/calmodulin-dependent protein kinase II improves contractility in human failing myocardium. *Circ. Res.* 107:1150–1161. 10.1161/CIRCRESAHA.110.220418 20814023

[B193] Srinivasan N. T. Schilling R. J. (2018). Sudden cardiac death and arrhythmias. *Arrhythm. Electrophysiol. Rev.* 7 111–117. 29967683 10.15420/aer.2018:15:2 PMC6020177

[B194] Stanger O. Aigner I. Schimetta W. Wonisch W. (2014). Antioxidant supplementation attenuates oxidative stress in patients undergoing coronary artery bypass graft surgery. *Tohoku J. Exp. Med.* 232 145–154. 10.1620/tjem.232.145 24573122

[B195] Stoica R. De Vos K. J. Paillusson S. Mueller S. Sancho R. M. Lau K. F. (2014). ER-mitochondria associations are regulated by the VAPB-PTPIP51 interaction and are disrupted by ALS/FTD-associated TDP-43. *Nat. Commun.* 5:3996. 10.1038/ncomms4996 PMC4046113 24893131

[B196] Sugiyama A. Aye N. N. Sawada N. Hashimoto K. (1999). Cariporide, a highly selective Na^+^/H^+^ exchange inhibitor, suppresses the reperfusion-induced lethal arrhythmias and “overshoot” phenomenon of creatine phosphate in situ rat heart. *J. Cardiovasc. Pharmacol.* 33 116–121. 10.1097/00005344-199901000-00017 9890405

[B197] Sugiyama S. Hattori M. Miyazaki Y. Nagai S. Ozawa T. (1985). The effect of verapamil on reperfusion arrhythmia in canine heart. *Jpn. Circ. J.* 49 1235–1242. 10.1253/jcj.49.1235 3831393

[B198] Sventzouri S. Nanas I. Vakrou S. Kapelios C. Sousonis V. Sfakianaki T. (2018). Pharmacologic inhibition of the mitochondrial Na^+^/Ca^2+^ exchanger protects against ventricular arrhythmias in a porcine model of ischemia-reperfusion. *Hellen. J. Cardiol.* 59 217–222. 10.1016/j.hjc.2017.12.009 29292245

[B199] Szabadkai G. Bianchi K. Várnai P. De Stefani D. Wieckowski M. R. Cavagna D. (2006). Chaperone-mediated coupling of endoplasmic reticulum and mitochondrial Ca2+ channels. *J. Cell Biol.* 175 901–911. 10.1083/jcb.200608073 17178908 PMC2064700

[B200] Takimoto E. Kass D. A. (2007). Role of oxidative stress in cardiac hypertrophy and remodeling. *Hypertension* 49 241–248. 10.1161/01.hyp.0000254415.31362.a7 17190878

[B201] Terentyev D. Györke I. Belevych A. E. Terentyeva R. Sridhar A. Nishijima Y. (2008). Redox modification of ryanodine receptors contributes to sarcoplasmic reticulum Ca2+ leak in chronic heart failure. *Circ. Res.* 103 1466–1472. 10.1161/circresaha.108.184457 19008475 PMC3274754

[B202] Testai L. Marino A. Piano I. Brancaleone V. Tomita K. Di Cesare Mannelli L. (2016). The novel H_2_S-donor 4-carboxyphenyl isothiocyanate promotes cardioprotective effects against ischemia/reperfusion injury through activation of mitoK_ATP_ channels and reduction of oxidative stress. *Pharmacol. Res.* 113(Pt A) 290–299. 10.1016/j.phrs.2016.09.006 27616550

[B203] Toda T. Kadono T. Hoshiai M. Eguchi Y. Nakazawa S. Nakazawa H. (2007). Na^+^/H^+^ exchanger inhibitor cariporide attenuates the mitochondrial Ca^2+^ overload and PTP opening. *Am. J. Physiol. Heart Circ. Physiol.* 293 H3517–H3523. 10.1152/ajpheart.00483.2006 17906113

[B204] Tränkmann P. Thiele R. Winnefeld K. Seliger K. (1999). Effect of administration of selenium and vitamin E on heart failure and ventricular arrhythmias in patients with acute myocardial infarct. *Med. Klin.* 94(Suppl. 3) 78–80. 10.1007/BF03042199 10554537

[B205] Tsai M. S. Huang C. H. Tsai C. Y. Chen H. W. Lee H. C. Cheng H. J. (2011). Ascorbic acid mitigates the myocardial injury after cardiac arrest and electrical shock. *Intens. Care Med.* 37 2033–2040. 10.1007/s00134-011-2362-6 21953354

[B206] Tsutsumi Y. Oshita S. Kawano T. Kitahata H. Tomiyama Y. Kuroda Y. (2001). Lidocaine and mexiletine inhibit mitochondrial oxidation in rat ventricular myocytes. *Anesthesiology* 95 766–770. 10.1097/00000542-200109000-00032 11575552

[B207] Turner D. M. Walker J. B. (1985). Relative abilities of phosphagens with different thermodynamic or kinetic properties to help sustain ATP and total adenylate pools in heart during ischemia. *Arch. Biochem. Biophys.* 238 642–651. 10.1016/0003-9861(85)90210-3 3994395

[B208] Ugdyzhekova D. S. Afanas’ev S. A. Lukavskaia I. A. Popov S. V. (2005). Cardiac effects of the class III antiarrhythmic drugs amiodarone and nibentan. *Fiziol. Cheloveka* 31 113–118. 16122045

[B209] Ullrich M. Aßmus B. Augustin A. M. Häbich H. Abeßer M. Martin Machado J. (2019). SPRED2 deficiency elicits cardiac arrhythmias and premature death via impaired autophagy. *J. Mol. Cell Cardiol.* 129 13–26. 10.1016/j.yjmcc.2019.01.023 30771306

[B210] Vásquez-Trincado C. García-Carvajal I. Pennanen C. Parra V. Hill J. A. Rothermel B. A. (2016). Mitochondrial dynamics, mitophagy and cardiovascular disease. *J. Physiol.* 594 509–525. 10.1113/jp271301 26537557 PMC5341713

[B211] Vazquez Ruiz de Castroviejo E. Munoz Bellido J. Lozano Cabezas C. Ramirez Moreno A. Guzman Herrera M. Tarabini Castellani A. (2005). Analysis of the frequency of cardiac arrhythmias and conduction disturbances from a health-care perspective. *Rev. Esp. Cardiol.* 58 657–665. 10.1016/s1885-5857(06)60252-1 15970121

[B212] Vázquez-Garza E. Bernal-Ramírez J. Jerjes-Sánchez C. Lozano O. Acuña-Morín E. Vanoye-Tamez M. (2020). Resveratrol prevents right ventricle remodeling and dysfunction in monocrotaline-induced pulmonary arterial hypertension with a limited improvement in the lung vasculature. *Oxid. Med. Cell Longev.* 2020:1841527. 10.1155/2020/1841527 PMC7023844 32089765

[B213] Verheule S. Sato T. Everett T. T. Engle S. K. Otten D. Rubart-von der Lohe M. (2004). Increased vulnerability to atrial fibrillation in transgenic mice with selective atrial fibrosis caused by overexpression of TGF-beta1. *Circ. Res.* 94 1458–1465. 10.1161/01.res.0000129579.59664.9d 15117823 PMC2129102

[B214] Violi F. Pastori D. Pignatelli P. Loffredo L. (2014). Antioxidants for prevention of atrial fibrillation: a potentially useful future therapeutic approach? A review of the literature and meta-analysis. *Europace* 16 1107–1116. 10.1093/europace/euu040 24706090

[B215] Vranka I. Penz P. Dukát A. (2007). Atrial conduction delay and its association with left atrial dimension, left atrial pressure and left ventricular diastolic dysfunction in patients at risk of atrial fibrillation. *Exp. Clin. Cardiol.* 12 197–201. 18651004 PMC2359612

[B216] Wai T. García-Prieto J. Baker M. J. Merkwirth C. Benit P. Rustin P. (2015). Imbalanced OPA1 processing and mitochondrial fragmentation cause heart failure in mice. *Science* 350:aad0116. 10.1126/science.aad0116 26785494

[B217] Wang C. Hu S. M. Xie H. Qiao S. G. Liu H. Liu C. F. (2015). Role of mitochondrial ATP-sensitive potassium channel-mediated PKC-ε in delayed protection against myocardial ischemia/reperfusion injury in isolated hearts of sevoflurane-preconditioned rats. *Braz. J. Med. Biol. Res.* 48 528–536. 10.1590/1414-431x20143876 25831209 PMC4470312

[B218] Wang S. Radhakrishnan J. Ayoub I. M. Kolarova J. D. Taglieri D. M. Gazmuri R. J. (2007). Limiting sarcolemmal Na+ entry during resuscitation from ventricular fibrillation prevents excess mitochondrial Ca2+ accumulation and attenuates myocardial injury. *J. Appl. Physiol.* 103 55–65. 10.1152/japplphysiol.01167.2006 17431086

[B219] Wang Y. G. Liu C. Z. Li Y. Z. Peng Y. Yan S. (2020). Cotreatments with Dex and Na(2)SeO(3) further improved antioxidant and anti-inflammatory protection of myocardial cells from I/R injury compared to their individual treatments. *Free Radic. Res.* 54 76–90. 10.1080/10715762.2019.1707198 31909644

[B220] Wehrens X. H. Lehnart S. E. Huang F. Vest J. A. Reiken S. R. Mohler P. J. (2003). FKBP12.6 deficiency and defective calcium release channel (ryanodine receptor) function linked to exercise-induced sudden cardiac death. *Cell* 113 829–840. 10.1016/s0092-8674(03)00434-3 12837242

[B221] Wehrens X. H. Lehnart S. E. Reiken S. R. Deng S. X. Vest J. A. Cervantes D. (2004). Protection from cardiac arrhythmia through ryanodine receptor-stabilizing protein calstabin2. *Science* 304 292–296. 10.1126/science.1094301 15073377

[B222] Wiersma M. van Marion D. M. S. Wüst R. C. I. Houtkooper R. H. Zhang D. Groot N. M. S. (2019). Mitochondrial dysfunction underlies cardiomyocyte remodeling in experimental and clinical Atrial fibrillation. *Cells* 8:1202. 10.3390/cells8101202 31590355 PMC6829298

[B223] Wiesmann T. Freitag D. Dersch W. Eschbach D. Irqsusi M. Steinfeldt T. (2017). Dantrolene versus amiodarone for cardiopulmonary resuscitation: a randomized, double-blinded experimental study. *Sci. Rep.* 7:40875. 10.1038/srep40875 28098197 PMC5241655

[B224] Wijffels M. C. Kirchhof C. J. Dorland R. Allessie M. A. (1995). Atrial fibrillation begets atrial fibrillation. A study in awake chronically instrumented goats. *Circulation* 92 1954–1968. 10.1161/01.cir.92.7.1954 7671380

[B225] Wilson D. N. Schacht S. E. Al-Nakkash L. Babu J. R. Broderick T. L. (2016). Resveratrol prevents pulmonary trunk remodeling but not right ventricular hypertrophy in monocrotaline-induced pulmonary hypertension. *Pathophysiology* 23 243–250. 10.1016/j.pathophys.2016.05.004 27374951

[B226] Wilting F. Kopp R. Gurnev P. A. Schedel A. Dupper N. J. Kwon O. (2020). The antiarrhythmic compound efsevin directly modulates voltage-dependent anion channel 2 by binding to its inner wall and enhancing mitochondrial Ca(2+) uptake. *Br. J. Pharmacol.* 177 2947–2958. 10.1111/bph.15022 32059260 PMC7279994

[B227] Wongcharoen W. Chen Y. C. Chen Y. J. Chen S. Y. Yeh H. I. Lin C. I. (2007). Aging increases pulmonary veins arrhythmogenesis and susceptibility to calcium regulation agents. *Heart Rhythm.* 4 1338–1349. 10.1016/j.hrthm.2007.06.023 17905341

[B228] Woodward B. Zakaria M. N. (1985). Effect of some free radical scavengers on reperfusion induced arrhythmias in the isolated rat heart. *J. Mol. Cell Cardiol.* 17 485–493. 10.1016/s0022-2828(85)80053-5 3928898

[B229] Wu H. Ye M. Liu D. Yang J. Ding J. W. Zhang J. (2019). UCP2 protect the heart from myocardial ischemia/reperfusion injury via induction of mitochondrial autophagy. *J. Cell Biochem.* 120 15455–15466. 10.1002/jcb.28812 31081966

[B230] Wu S. Lu Q. Wang Q. Ding Y. Ma Z. Mao X. (2017). Binding of FUN14 domain containing 1 With Inositol 1,4,5-trisphosphate receptor in mitochondria-associated endoplasmic reticulum membranes maintains mitochondrial dynamics and function in hearts in vivo. *Circulation* 136 2248–2266. 10.1161/circulationaha.117.030235 28942427 PMC5716911

[B231] Wu Y. Rasmussen T. P. Koval O. M. Joiner M. L. Hall D. D. Chen B. (2015). The mitochondrial uniporter controls fight or flight heart rate increases. *Nat. Commun.* 6:6081. 10.1038/ncomms7081 PMC4398998 25603276

[B232] Xiao D. Gu Z. L. Qian Z. N. (1993). Effects of quercetin on platelet and reperfusion-induced arrhythmias in rats. *Zhongguo Yao Li Xue Bao* 14 505–508. 8010047

[B233] Xie A. Song Z. Liu H. Zhou A. Shi G. Wang Q. (2018). Mitochondrial Ca^2+^ influx contributes to arrhythmic risk in nonischemic cardiomyopathy. *J. Am. Heart Assoc.* 7:7805. 10.1161/JAHA.117.007805 29627768 PMC6015427

[B234] Xie J. R. Yu L. N. (2007). Cardioprotective effects of cyclosporine A in an in vivo model of myocardial ischemia and reperfusion. *Acta Anaesthesiol. Scand.* 51 909–913. 10.1111/j.1399-6576.2007.01342.x 17578461

[B235] Xie W. Santulli G. Reiken S. R. Yuan Q. Osborne B. W. Chen B. X. (2015). Mitochondrial oxidative stress promotes atrial fibrillation. *Sci. Rep.* 5:11427. 10.1038/srep11427 PMC4501003 26169582

[B236] Xu D. Murakoshi N. Igarashi M. Hirayama A. Ito Y. Seo Y. (2012). PPAR-γ activator pioglitazone prevents age-related atrial fibrillation susceptibility by improving antioxidant capacity and reducing apoptosis in a rat model. *J. Cardiovasc. Electrophysiol.* 23 209–217. 10.1111/j.1540-8167.2011.02186.x 21954843

[B237] Xu S. Wang P. Zhang H. Gong G. Gutierrez Cortes N. Zhu W. (2016). CaMKII induces permeability transition through Drp1 phosphorylation during chronic β-AR stimulation. *Nat. Commun.* 7:13189. 10.1038/ncomms13189 PMC5067512 27739424

[B238] Yang K. C. Kyle J. W. Makielski J. C. Dudley S. C. Jr. (2015). Mechanisms of sudden cardiac death: oxidants and metabolism. *Circ. Res.* 116 1937–1955. 10.1161/circresaha.116.304691 26044249 PMC4458707

[B239] Yu Z. Chen R. Li M. Yu Y. Liang Y. Han F. (2018). Mitochondrial calcium uniporter inhibition provides cardioprotection in pressure overload-induced heart failure through autophagy enhancement. *Int. J. Cardiol.* 271 161–168. 10.1016/j.ijcard.2018.05.054 29803339

[B240] Zhang J. Corciulo C. Liu H. Wilder T. Ito M. Cronstein B. (2017). Adenosine A(2a) receptor blockade diminishes Wnt/β-Catenin signaling in a murine model of bleomycin-induced dermal fibrosis. *Am. J. Pathol.* 187 1935–1944. 10.1016/j.ajpath.2017.05.005 28667836 PMC5809334

[B241] Zhang C. Deng Y. Lei Y. Zhao J. Wei W. Li Y. (2017). Effects of selenium on myocardial apoptosis by modifying the activity of mitochondrial STAT3 and regulating potassium channel expression. *Exp. Ther. Med.* 14 2201–2205. 10.3892/etm.2017.4716 28962142 PMC5609099

[B242] Zhang L. Deng T. Sun Y. Liu K. Yang Y. Zheng X. (2008). Role for nitric oxide in permeability of hippocampal neuronal hemichannels during oxygen glucose deprivation. *J. Neurosci. Res.* 86 2281–2291. 10.1002/jnr.21675 18381763

[B243] Zhang T. Maier L. S. Dalton N. D. Miyamoto S. Ross J. Jr. Bers D. M. (2003). The deltaC isoform of CaMKII is activated in cardiac hypertrophy and induces dilated cardiomyopathy and heart failure. *Circ. Res.* 92 912–919. 10.1161/01.res.0000069686.31472.c5 12676814

[B244] Zhang Y. Shimizu H. Siu K. L. Mahajan A. Chen J. N. Cai H. (2014). NADPH oxidase 4 induces cardiac arrhythmic phenotype in zebrafish. *J. Biol. Chem.* 289 23200–23208. 10.1074/jbc.m114.587196 24962575 PMC4132817

[B245] Zhao W. Frank K. F. Chu G. Gerst M. J. Schmidt A. G. Ji Y. (2003). Combined phospholamban ablation and SERCA1a overexpression result in a new hyperdynamic cardiac state. *Cardiovasc. Res.* 57 71–81. 10.1016/s0008-6363(02)00609-0 12504816

[B246] Zhou L. Solhjoo S. Millare B. Plank G. Abraham M. R. Cortassa S. (2014). Effects of regional mitochondrial depolarization on electrical propagation: implications for arrhythmogenesis. *Circ. Arrhythm. Electrophysiol.* 7 143–151. 10.1161/circep.113.000600 24382411 PMC4001739

[B247] Zhu J. Rebecchi M. J. Tan M. Glass P. S. Brink P. R. Liu L. (2010). Age-associated differences in activation of Akt/GSK-3beta signaling pathways and inhibition of mitochondrial permeability transition pore opening in the rat heart. *J. Gerontol. A Biol. Sci. Med. Sci.* 65 611–619. 10.1093/gerona/glq035 20427381

[B248] Zima A. V. Blatter L. A. (2006). Redox regulation of cardiac calcium channels and transporters. *Cardiovasc. Res.* 71 310–321. 10.1016/j.cardiores.2006.02.019 16581043

[B249] Zissimopoulos S. Docrat N. Lai F. A. (2007). Redox sensitivity of the ryanodine receptor interaction with FK506-binding protein. *J. Biol. Chem.* 282 6976–6983. 10.1074/jbc.m607590200 17200109

[B250] Zulian A. Sileikytë J. Petronilli V. Bova S. Dabbeni-Sala F. Cargnelli G. (2011). The translocator protein (peripheral benzodiazepine receptor) mediates rat-selective activation of the mitochondrial permeability transition by norbormide. *Biochim. Biophys. Acta* 1807 1600–1605. 10.1016/j.bbabio.2011.08.007 21889488

[B251] Zulkifly H. Lip G. Y. H. Lane D. A. (2018). Epidemiology of atrial fibrillation. *Int. J. Clin. Pract.* 72:e13070. 10.1111/ijcp.13070 29493854

[B252] Zuo S. Li L. L. Ruan Y. F. Jiang L. Li X. Li S. N. (2018). Acute administration of tumour necrosis factor-α induces spontaneous calcium release via the reactive oxygen species pathway in atrial myocytes. *Europace* 20 1367–1374. 10.1093/europace/eux271 29045723

